# Analysis of the Genome and Metabolome of Marine Myxobacteria Reveals High Potential for Biosynthesis of Novel Specialized Metabolites

**DOI:** 10.1038/s41598-018-34954-y

**Published:** 2018-11-09

**Authors:** Jamshid Amiri Moghaddam, Max Crüsemann, Mohammad Alanjary, Henrik Harms, Antonio Dávila-Céspedes, Jochen Blom, Anja Poehlein, Nadine Ziemert, Gabriele M. König, Till F. Schäberle

**Affiliations:** 1 Institute for Pharmaceutical Biology, University of Bonn, Bonn, Germany; 2 Department of Microbiology and Biotechnology, University of Tübingen, Tübingen, Germany; 3 German Center for Infection Research (DZIF) Partner Site Cologne/Bonn, Bonn, Germany; 4 Institute for Insect Biotechnology, Justus Liebig University Giessen, Giessen, Germany; 5 Bioinformatics and Systems Biology, Justus Liebig University Giessen, Giessen, Germany; 6 Department of Genomics and Applied Microbiology and Göttingen Genomics Laboratory, Georg-August-University Göttingen, Göttingen, Germany; 7 Department of Bioresources of the Fraunhofer Institute for Molecular Biology and Applied Ecology, Giessen, Germany

## Abstract

Comparative genomic/metabolomic analysis is a powerful tool to disclose the potential of microbes for the biosynthesis of novel specialized metabolites. In the group of marine myxobacteria only a limited number of isolated species and sequenced genomes is so far available. However, the few compounds isolated thereof so far show interesting bioactivities and even novel chemical scaffolds; thereby indicating a huge potential for natural product discovery. In this study, all marine myxobacteria with accessible genome data (n = 5), including *Haliangium ochraceum* DSM 14365, *Plesiocystis pacifica* DSM 14875, *Enhygromyxa salina* DSM 15201 and the two newly sequenced species *Enhygromyxa salina* SWB005 and SWB007, were analyzed. All of these accessible genomes are large (~10 Mb), with a relatively small core genome and many unique coding sequences in each strain. Genome analysis revealed a high variety of biosynthetic gene clusters (BGCs) between the strains and several resistance models and essential core genes indicated the potential to biosynthesize antimicrobial molecules. Polyketides (PKs) and terpenes represented the majority of predicted specialized metabolite BGCs and contributed to the highest share between the strains. BGCs coding for non-ribosomal peptides (NRPs), PK/NRP hybrids and ribosomally synthesized and post-translationally modified peptides (RiPPs) were mostly strain specific. These results were in line with the metabolomic analysis, which revealed a high diversity of the chemical features between the strains. Only 6–11% of the metabolome was shared between all the investigated strains, which correlates to the small core genome of these bacteria (13–16% of each genome). In addition, the compound enhygrolide A, known from *E*. *salina* SWB005, was detected for the first time and structurally elucidated from *Enhygromyxa salina* SWB006. The here acquired data corroborate that these microorganisms represent a most promising source for the detection of novel specialized metabolites.

## Introduction

Microorganisms are well known for their ability to produce so called secondary or rather specialized metabolites, of which many have proven to have important medical, biotechnological, agricultural, and nutritional applications^1^. In recent years, genome mining has led to a paradigm shift in natural products research as biosynthetic gene clusters (BGCs), regions of the genome that encode for the production of natural products, can be identified and characterized from the genomes of many microorganisms^2^. A BGC represents both, a biosynthetic and an evolutionary unit, which can be identified using genome mining software tools like antiSMASH^3^. This sequence-based approach increases the chance for discovery of new metabolites by identifying the talented microbes using genome sequence analysis and subsequent characterization of the *in silico* identified BGCs^4^. The comprehensive biosynthetic potential, including silent clusters, rather than what is currently expressed and apparent in the lab, is shown. Combined with a metabolomic approach, using high resolution mass spectrometry and molecular networking, rediscovery of known metabolites can be avoided at a very early stage of the discovery process through dereplication^5,6^, and simultaneously, discovery of novel natural products can be streamlined through optimization of culture conditions^7^.

Marine environments, holding 95% of the earth’s biosphere, have come into the focus for natural product discovery as a consequence of the emergence of antimicrobial resistance, boosted by the limitations in novel drug developments from the usual producers of terrestrial environments^4^. Myxobacteria are a group of Deltaproteobacteria, which have been first discovered from soil since 1809. These organisms were thought to be occurring exclusively in terrestrial environments until Iizuka *et al*. reported in 1998 the isolation of myxobacteria from a marine environment^8,9^. Terrestrial myxobacteria have been well investigated over the past three decades, which resulted in more than 100 natural product scaffolds and approximately 600 structural derivatives with a broad range of biological activities^10^. However, to date, only 10 obligatory marine myxobacterial strains, which need sea-like conditions in order to grow, have been isolated and from them, only seven groups of natural products have been identified, including enhygrolides, enhygromic acid, haliamide, haliangicins, salimabromide, salimyxins, and triterpenoid sterols (Fig. S1)^11–14^. The lack of more marine myxobacterial isolates and natural products is mainly due to the difficulties in isolation and cultivation of these bacteria^15^.

Here, we conducted comparative genomic analysis of the five marine myxobacteria for which genomes are publicly available, thereunder two newly sequenced strains from our lab. This analysis was carried out in order to compare the similarities and differences in the biosynthesis of specialized metabolites in marine myxobacteria. We report the distribution and similarity within the existing BGCs in the genomes, revealing the uniqueness and variability of BGCs harbored by these bacteria. Furthermore, metabolomes of the marine myxobacterial strains were analyzed and compared using mass spectral networking, to evaluate if the trends from genome analysis are translatable into actual metabolite profiles.

## Material and Methods

### Strains and isolates

The marine myxobacterial strains *Enhygromyxa salina* SWB005, SWB006 and SWB007 were obtained from the strain collection of the Institute for Pharmaceutical Biology, University of Bonn, Bonn, Germany. These strains have been isolated from marine sediments, which originated from beach areas of Santa Barbara, U.S. (*E*. *salina* SWB005), of Borkum, Germany (*E*. *salina* SWB006) and Prerow, Germany (*E*. *salina* SWB007)^16,17^. *Enhygromyxa salina* DSM 15201 (formerly named *E*. *salina* SMP-6) and *Plesiocystis pacifica* DSM 14875 (type strain, formerly named *P*. *pacifica* SIR-1) were obtained from the German Collection of Microorganisms and Cell Cultures (DSMZ). Those strains have been isolated from coastal sands (*E*. *salina* DSM 15201) and semi-dried seagrass (*P*. *pacifica* DSM 14875) of Japanese coasts^18^. A schematic workflow is given in the supplementary (Fig. S2), indicating which strains underwent which cultivation, processing, and analysis during this study.

### Genome sequencing and assembly

The genomic DNA of *E*. *salina* SWB005 and SWB007 was isolated as described before^19^. In brief, fruiting bodies, which appeared after several days of fermentation in ASW-VY/4 liquid medium (see cultivation for details), were harvested. DNA was isolated using the GenElute™ Bacterial Genomic DNA Kit (Sigma-Aldrich). Illumina shotgun paired-end sequencing libraries were generated and sequenced on a MiSeq instrument (Illumina, San Diego, CA, USA). Paired-end reads were combined using the Spades assembler v3.10, yielding initial sequence contigs^20^. After filtering contigs smaller than 500 bp, the remaining contigs were determined with Quast^21^. Genome completeness was estimated using CheckM^22^ and compared to the published genome data of *E*. *salina* DSM15201. The resulting genomes have been deposited at NCBI GenBank with the accession numbers PVNK00000000 (*E*. *salina* SWB005) and PVNL00000000 (*E*. *salina* SWB007)^19^. The genome sequences of *E*. *salina* DSM 15201, *P*. *pacifica* DSM 14875 and *Haliangium ochraceum* DSM 14365 were obtained from NCBI GenBank, accession numbers are JMCC00000000, ABCS00000000 and CP001804^23^, respectively.

### Genome alignment and annotation

To ease the comparative study of the draft genomes, Mauve Contig Mover (MCM)^24^ was used to order and/or reverse the contigs and align the other draft genomes relative to the *E*. *salina* SWB007 draft genome. FASTA files were used as input and the reordered FASTA files of the mauve output data were used for further analysis. Coding sequences of the reordered contigs were determined by using the RAST prokaryotic genome annotation server^25^. Therefore, the genetic code 11, which is used by most bacteria, was used in classic RAST and the options “automatically fix errors”, “fix frame shifts”, “build metabolic model” and “backfill gaps” were selected. To obtain the putative pathways of terpenoid buliding blocks, KEGG maps of the terpene backbone biosynthesis and degradation pathways of leucine, isoleucine and valine were compiled using RAST as hierarchical trees^25,26^. All reactions for a given cellular process with links to the KEGG map were visualized with annotated proteins, which putatively catalyze the reaction^25^.

### Genome comparison

The EDGAR 2.2 genomic pipeline was used for genome comparison^27^. Therefore, the RAST-annotated GenBank files were uploaded to EDGAR and the core genome, orthologous genes and singletons were identified. Visualization was done using a Venn diagram; core genome size and gene numbers in every subset of the dispensable genomes were indicated. To visualize the drop of the core genome size and the increase of the pan genome with the introduction of each genome, a core vs. pan plot of the genomes was generated. To compare the gene order and co-localization of genes in the different genomes, a synteny plot was generated. *Haliangium ochraceum* DSM 14875 was omitted from the synteny plot analysis; due to the fact that not enough conserved regions in comparison with the other strains exist.

A phylogenetic tree of the investigated marine myxobacteria was constructed based on a linear combination of multiple alignments of the nucleotide sequences of orthologous genes in the core genome. The alignments were created using MUSCLE^28^, and the PHYLIP^29^ implementation of the neighbor-joining algorithm was used to deduce the tree. For a deeper qualitative comparison between the genomes, the average amino acid identity (AAI) and average nucleotide identity (ANI) matrixes of all conserved genes in the core genome were computed by the BLAST algorithm and visualized as heat maps. In silico DNA-DNA hybridization (isDDH) was performed based on identities/HSP length formula using the DSMZ GGDC service tool^30^. The CGView Comparison Tool (CCT) was used to create a graphical map of the BLAST results comparison of the available genomes to the genome of *E*. *salina* SWB007^31^.

### Prediction of specialized metabolites biosynthetic gene clusters

Biosynthetic gene clusters (BGCs) for specialized metabolites were identified using AntiSMASH v4^3^ with the ClusterFinder algorithm; no additional options were applied in the analysis. The distribution of all identified BGCs of the AntiSMASH analysis was visualized in a circular chord diagram using Circos table viewer, whereby the putative BGCs were not considered^32^. A similarity network of the BGCs among different genomes was obtained using a modified Pfam domain similarity metric implemented in BigScape^33,34^. A cut-off of 0.75 was used for the analysis^34^. Additional screening for resistance markers and potential antibiotic targets was performed using the ARTS webserver^35^ and clusters positive for known resistance markers and duplicated essential genes were subsequently annotated in the final similarity network using Cytoscape 3.6.1. This was performed using custom python scripts to collect and format the BigScape similarity tables into gml format (https://github.com/malanjary-ut/helperscripts). The similarity network file is available at NDEx^36^ (http://doi.org/10.18119/N9F30V). The fraction of the genomes with a shared BGC that is devoted to specialized metabolism was aligned using EDGAR regional alignment to enable comparison of the similar gene clusters^27^.

#### Cultivation, extraction, and isolation

All bacteria were grown in ASW-VY/4 medium (1 L contains 75% artificial sea water (ASW), 25 mL of a 10% yeast suspension, trace elements solution and vitamin B_12_ filled up to the final volume with milli-Q water. Standard artificial sea water contains KBr (0.2 g/L), NaCl (46.96 g/L), MgCl_2_-hexahydrate (21.22 g/L), CaCl_2_-dihydrate (2.94 g/L), KCl (1.32 g/l), SrCl_2_-hexahydrate (0.08 g/L), Na_2_SO_4_ (7.84 g/L), NaHCO_3_ (0.38 g/L), H_3_B0_3_ (0.06 g/L). Trace element solution: ZnCl_2_ (20 mg/L), MnCl_2_ x 4 H_2_O (100 mg/L), H_3_BO_3_ (10 mg/L), CuSO_4_ (10 mg/L), CoCl_2_ (20 mg/L), SnCl_2_ x 2 H_2_O (5 mg/L), LiCl (5 mg/L), KBr (20 mg/L),) KI (20 mg/L, Na_2_MoO_4_ x 2 H_2_O (10 mg/L) and Na_2_-EDTA x 2 H_2_O (5.2 g/L) in distilled water and sterilized by filtration. Two 100 mL precultures, containing visible fruiting bodies, were used to inoculate 1 L ASW-VY/4 medium, respectively. The cultures were shaken on a rotary shaker at 140 rpm for 14 days at 30 °C. Adsorber resin Sepabeads® SP207 (Supelco, 20 g/L) was added to the cultures 48 hours before extraction. Bacterial pellet and adsorber resin were separated from the medium with a filter (pore size 2) and extracted with approx. 500 mL acetone until the organic phase became uncolored. After the organic solvent was evaporated under vacuum conditions, the residue was redissolved in 100 mL aqueous methanol (60%) and extracted seven times with 100 mL dichloromethane. Crude lipophilic dichlormethane extracts were thus obtained. The extracts were further separated via RP_18_ Solid-Phase-Extraction (SPE) utilizing Bakerbond SPE Silica 1000 mg/6 mL columns and reduced pressure in a Bakerbound vacuum chamber. Thereby, a stepwise elution process with respectively 30 mL of petroleum ether, dichloromethane, acetone ethyl acetate, and methanol was employed.

For the isolation of enhygrolide A the myxobacterial strain *E*. *salina* SWB006 was cultivated in a 30 L stirred bioreactor using 20 L ASW-VY/4 medium containing 10 g/L of the adsorber resin Amberlite® XAD16N (Dow Chemical Company). The culture was grown at 28 °C, an airflow of 5 L/min and stirring at 200 rpm. After 120 h, the biomass and the adsorber resin were harvested by centrifugation and extracted with acetone and methanol until the organic phase got uncolored. The acetone phase was lyophilized and the residual 824 mg crude acetone extract was solved in acetone and adsorbed at 40 g Celite® 545 material.

This material was fractionated on a 12 g NP Silica 40 µm Reveleris® Flash cartridge by automatized Chromatography Systems REVELERIS® X2 Flash with integrated evaporative light scattering (ELSD)/ UV-Vis detection. A stepwise gradient solvent system of increasing polarity and a flow rate of 30 mL/min was used, starting with 100% hexane for 4.0 min to 100% CH2Cl2 within 6.0 min and hold for 3.0 min at 100% CH2Cl2. The gradient was changed then within 13.0 min to 100% EtOAc. Finally, the gradient was changed within 5.0 min to 20% MeOH and the cartridge was washed for additional 15 min under these conditions. According to the measured ELSD and UV signals at λ = 290, 320, and 350 nm the crude extract was separated into 18 fractions. Fraction ten, tR: 13–14 min yielded 2.0 mg of Enhygrolide A. The structure was confirmed by comparison of ^1^H- and ^13^C-NMR and HRESI-MS data with literature values^15^.

#### Enhygrolide A

white powder; ^1^H and ^13^C NMR data (see Table S2), HRESI-MS *m*/*z* = 357.1464 [M + H]^+^ (calcd. for C_22_H_22_NaO_3_, *m*/*z:* 357.1461, 3.35 Δppm).

### HPLC-MS/MS analysis

Samples were analyzed by HPLC-MS/MS on a micrOTOF-Q mass spectrometer (Bruker) with ESI-source coupled with a HPLC Dionex Ultimate 3000 (Thermo Scientific) using a Zorbax Eclipse Plus C18 1.8 µm column, 2.1 × 50 mm (Agilent). The column temperature was 45 °C. MS data were acquired over a range from 100–3000 m/z in positive mode. Auto MS/MS fragmentation was achieved with rising collision energy (35–50 keV over a gradient from 500–2000 m/z) with a frequency of 4 Hz for all ions over a threshold of 100. UHPLC starting conditions with 90% H_2_O containing 0.1% acetic acid as mobile phase were kept isocratic for 0.5 min. Followed by a gradient solvent system to 100% acetonitrile (0.1% acetic acid) within 4 min. 2 µl of sample solution was injected to a flow of 0.8 ml/min. All MS/MS data were converted to.mzXML format, transferred to the GNPS server (gnps.ucsd.edu) (Wang *et al*., 2016) and uploaded to massive.ucsd.edu as dataset MSV000082831. Molecular networking was performed based on the GNPS data analysis workflow using the spectral clustering algorithm^37^.

### Molecular networking

For the molecular network analysis, all nodes that contained ions from blank medium were removed. A molecular network was created by the online workflow at GNPS^38^ using the spectra with a minimum of four fragment ions and by merging all identical spectra into nodes, representing parent masses. Compounds with similar fragmentation patterns are connected by edges, displaying molecular families with similar structural features. The data was filtered by removing all MS/MS peaks within +/−17 Da of the precursor m/z. MS/MS spectra were window filtered by choosing only the top 6 peaks in the +/−50 Da window throughout the spectrum. The resulting data were then clustered by MS-Cluster with a parent mass tolerance of 0.02 Da and a MS/MS fragment ion tolerance of 0.02 Da to create consensus spectra. Further, consensus spectra that contained less than 2 spectra were discarded. A network was then created where edges were filtered to have a cosine score above 0.5 and more than 4 matched peaks. Further edges between two nodes were kept into the network if and only if each of the nodes appeared in each other’s respective top 10 most similar nodes. The spectra in the network were then searched against GNPS’ spectral libraries. The library spectra were filtered in the same manner as the input data including analog search. All matches kept between network spectra and library spectra were required to have a score above 0.5 and at least 4 matched peaks. The network was visualized via Cytoscape 3.6.1. The molecular network file is available at NDEx (http://doi.org/10.18119/N9988C). Additionally, the molecular networking job is available at the GNPS server (https://gnps.ucsd.edu/ProteoSAFe/status.jsp?task=c90080f8763a4920bdf8117f64792e4c). A list of all the bioinformatics tools used to create the results with some general requirements is given in the supplementary information.

## Results

### General characteristics of marine myxobacterial genomes

Five draft genomes are currently available from obligatory marine myxobacteria: *Plesiocystis pacifica* DSM 14875, *Haliangium ochraceum* DSM 14365, *Enhygromyxa salina* DSM 15201, and, related to the latter one, *Enhygromyxa salina* SWB005 and *Enhygromyxa salina* SWB007, of which the last two were recently sequenced from our working group^19^ (Table 1). The quality of the draft genomes differs and the number of contigs varies between 1 for *H*. *ochraceum* DSM14365 to 330 for *E*. *salina* DSM 15201. However, all strains possess large genomes ranging from 9 to 10.6 Mbp. Like in terrestrial myxobacteria, the GC content is rather high, i.e. between 67 and 71% and the number of predicted gene coding sequences (CDS) is around 7,000–8,500, which is in accordance to the large genome size of these strains.

**Table 1:** General characteristics of available marine myxobacterial genomes.

Lineage	Bacteria; Proteobacteria; Deltaproteobacteria; Myxococcales				
Species	*Enhygromyxa salina*			*Plesiocystis pacifica*	*Haliangium ochraceum*
Strain^a^	SWB007	SWB005	DSM 15201	DSM 14875	DSM 14365
Genome size (Mbp)	10.6	9.0	10.4	10.6	9.4
GC Content	68.1	69.5	67.4	70.7	69.5
Number of Contigs	192	312	330	237	1
CDS	8293	7054	8178	8447	7032

^a^The accession numbers are given in the Material and Methods section.

**Figure 1:**
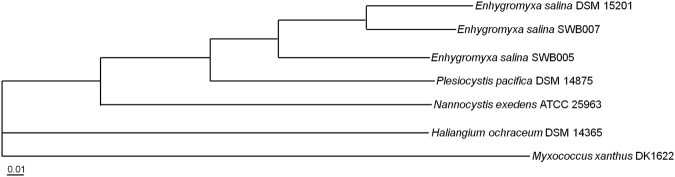
Phylogenetic tree of selected marine myxobacteria. Available genomes of marine myxobacteria were used to build the tree based on nucleotide sequence alignment of the core genomes. The closely related halophilic strain *Nannocystis exedens* ATCC 25963, as well as the terrestrial *Myxococcus xanthus* DK1622, which represents the outgroup, were included. Tree for 7 genomes, build out of a core of 645 genes per genome, 4515 in total. The core has 838,246 bp per genome, 5,867,722 in total. The tree topology was evaluated in 500 bootstrap iterations and showed a branch conservation of 100%.

A phylogenetic tree of marine myxobacteria was constructed based on a nucleotide sequence alignment of the core genomes (see below) (Fig. 1). The *E*. *salina* strains belong to the order of Myxococcales and the *P*. *pacifica* DSM 15201 type strain is the closest relative to the *E*. *salina* clade. They are part of the *Nannocystaceae* family. However, the first isolated marine myxobacterium *H*. *ochraceum* DSM 14365^T^ belongs to the family of *Kofleriaceae* and the core genome of this strain is distinct from the other marine myxobacteria (see below).

### Genome comparison

A synteny plot of the reciprocal best blast hits of all CDS within the contiguous contigs was constructed using the EDGAR pipeline. The genome of *E*. *salina* SWB007 was chosen as reference for synteny analysis, because (i) it is bigger in size, thereby the chance to cover genomic parts of the other strains is higher, (ii) it is of high quality, and (iii) due to the high relationship between the genera *Enhygromyxa* and *Plesiocystis*, which excludes *Haliangium* as reference. According to the synteny plot, there are many CDS located in different positions compared to the reference genome of *E*. *salina* SWB007. However, there is still rather good synteny of orthologous genes within the areas that reside inside contig boundaries of *E*. *salina* SWB007 and the genomes of *E*. *salina* SWB005 and DSM 15201, as well as *P*. *pacifica* DSM 14875. The latter showed slightly lower synteny (Fig. S3A). This result indicates a low degree of genome divergence within these marine myxobacteria. On the nucleotide level, *E*. *salina* SWB007 and DSM 15201 are highly similar, while the identity ratio of *E*. *salina* SWB005 is slightly lower and further decreases for *P*. *pacifica* DSM 14875 and *H*. *ochraceum* DSM 15365, respectively (Fig. S3B).

**Figure 2:**
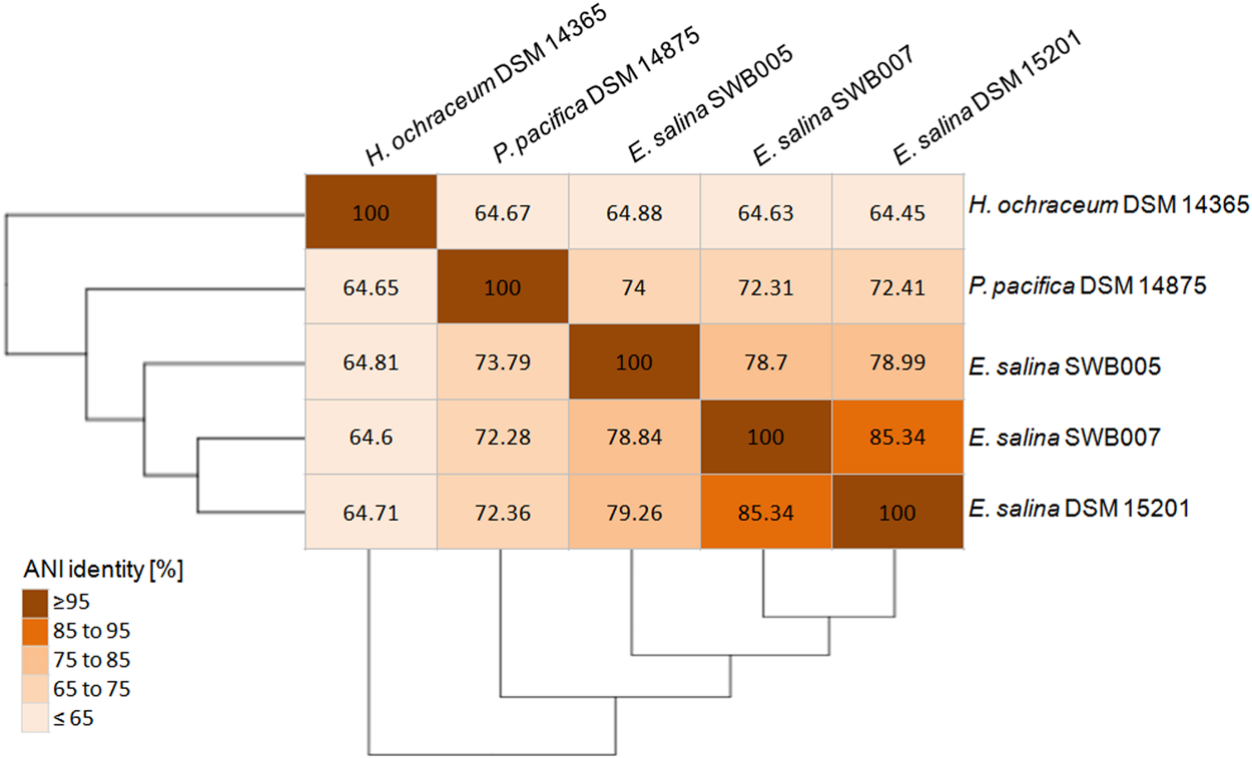
Average Nucleotides Identity (ANI) matrix of the available marine myxobacteria genomes. All values are given in percent.

Based on in silico parameters which determine if genomes belong to the same species (*i*.*e*. ANI value ≥ 96%, isDDH value ≥ 70%, and difference in G + C content ≤ 1%)^30,39^, both strains, *E*. *salina* SWB005 as well as SWB007, can be considered as a distinct new species of the genus *Enhygromyxa*. The ANI value between *E*. *salina* SWB007 and *E*. *salina* DSM 15201 is 85% with an isDDH value of 29% and a G + C difference of 0.7%. The ANI values between *E*. *salina* SWB005 and the other *E*. *salina* strains is 79% with an isDDH value of 23% and a G + C difference of more than 1% (Figs 2 and S4). On the amino acid level, all *E*. *salina* strains and *P*. *pacifica* DSM 14875 show 74.7–92.7% average amino acid identity (AAI) between each other (Fig. S5). Therefore, the orthologous genes in these strains probably perform the same functional roles. However, the function of the orthologous genes in *H*. *ochraceum* DSM 14365 is more uncertain, since the AAI is only 48% towards other strains (Fig. S5).

**Figure 3:**
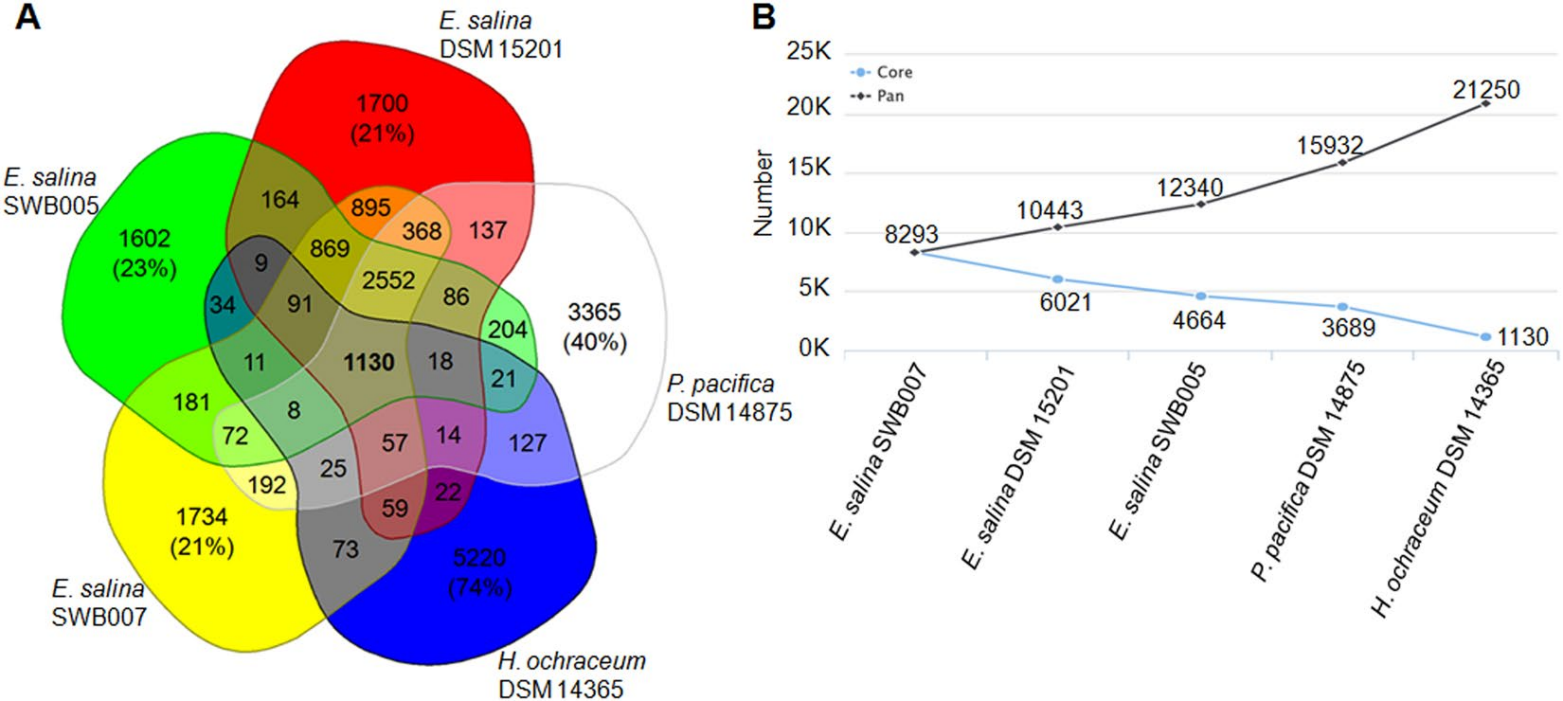
(**A**) Venn diagram of the CDS counts in different subsets of the genomes (singletons are given as percentage of the respective genome). (**B**) Core vs. pan genome plot of the genomes.

In order to obtain further insights into the degree of similarity between the analyzed genomes, the number of core genes, as well as of singletons was determined. (Fig. 3A). Even for the most closely related strains investigated here, *i*.*e*. the *E*. *salina* strains, more than 1600 CDS represent singletons, which is equivalent to 21–23% of each genome (Fig. 3A). This value duplicates if the next further relative, *i*.*e*. *P*. *pacifica* DSM 14875 is considered, since this strain has 3365 singletons (equivalent to ~40% of the genome). *H*. *ochraceum* DSM14365 has 5220 (equivalent to 74% of the genome) singletons (Fig. 3A). The core genome of these marine myxobacteria consists of 1130 CDS. This relatively low number, equivalent to 13–16% of the CDSs per strain, is due to the inclusion of the more distantly related *H*. *ochraceum* DSM 14365 genome to the analysis. For comparison, the core genome of six *Myxococcus* genomes, including 4 different species and three *M*. *xanthus* strains consists of 4,693 CDS. This accounts for 56.6–63% of the CDSs in each genome^40^. If only the *E*. *salina* strains are considered, they have > 4600 CDSs in their core genome, and inclusion of *P*. *pacifica* DSM 14875 in the analysis results in a core genome of >3600 CDSs (Fig. 3B). Hence, the pan genome increases by about 2000 CDSs by every additional *E*. *salina* strain. If the other marine myxobacteria are included, the pan genome increases further by almost 3500 CDSs of *P*. *pacifica* DSM 14875 and by 5000 CDSs of *Haliangium ochraceum* DSM14365, respectively (Fig. 3B).

### Analysis of specialized metabolite biosynthetic gene clusters in the genomes

In order to estimate the potential of the strains for the production of specialized metabolites, the genomes were screened *in silico* for the presence of biosynthetic gene clusters (BGCs) putatively coding for the production of such compounds^3^. All organisms investigated have a high variety of BGCs in their genomes, *i*.*e*. 30–46 BGCs were identified in each strain (Table 2). These numbers even doubled, if a more general cluster finder algorithm was applied to estimate the cluster boundaries (assigning putative BGCs) based on frequencies of locally encoded protein domains detected by Pfam^3^. In terms of novel metabolites, the numbers of identified BGCs by AntiSMASH which had similarities to known BGCs from the MIBiG database^41^ were counted (Table 2). 10–11 BGCs of each *E*. *salina* strain, 5 from *P*. *pacifica* DSM 14875, and 17 from *H*. *ochraceum* DSM 14365 matched partly or completely to validated gene clusters.

**Table 2:** Overview of predicted biosynthetic gene clusters (BGCs)^a^.

BGCs	E. salina SWB007	E. salina SWB005	E. salina DSM 15201	P. pacifica DSM 14875	H. ochraceum DSM 14365
Total^b^	80	56	77	76	62
Predicted by antiSMASH	46	40	38	34	30
Predicted putative	34	16	39	42	32
MIBiG hits	11	10	10	5	17
Known resistance model hits	9	7	13	4	4

^a^The number of BGCs predicted by antiSMASH and of the antibiotic-related BGCs predicted by ARTS is given. ^b^ Sum of the BGCs predicted by antiSMASH, also considering the putative ones.

**Figure 4:**
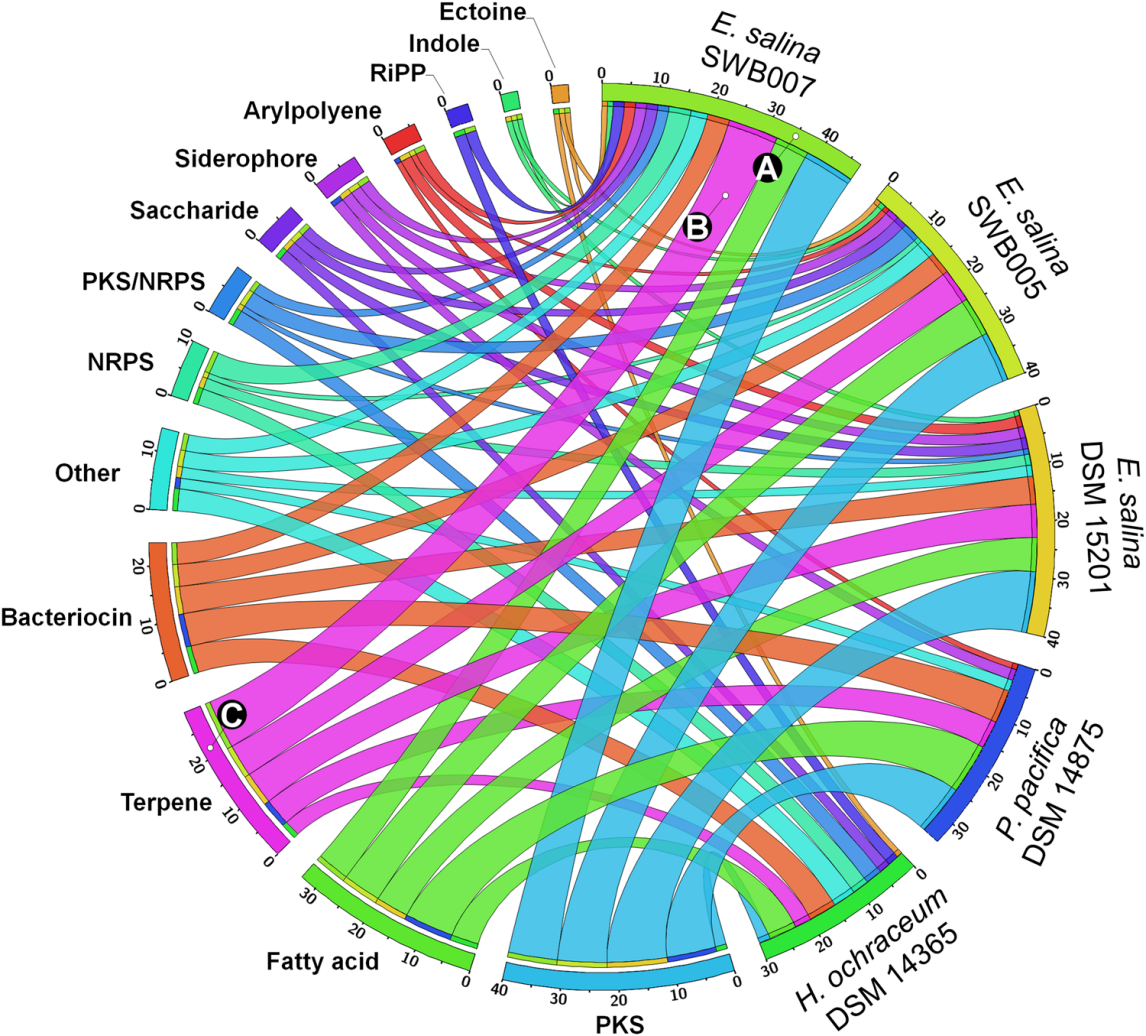
Distribution of different BGC types in the five genomes. Genomes (first) and BGC types (second) are segmented in descending order of the BGCs counts. (**A**) Color code of the respective strain and number of BGCs in each strain. (**B**) ribbon color is set to respective BGC type. (**C)** BGC color code.

Analyzing the classes of metabolites predicted from the identified BGCs, revealed that PKs (2–11 per strain), fatty acids (5–9), and terpenes (3–9) represent the majority of predicted specialized metabolites, followed by bacteriocins (4–6). NRPs (0–4) and mixed PK/NRPs (0–3) are less common (Fig. 4). However, it should be noted that because draft genome sequences were analyzed, big BGCs such as PKS and NRPS can be split across contigs and the real number of BGCs might be overestimated.

**Figure 5:**
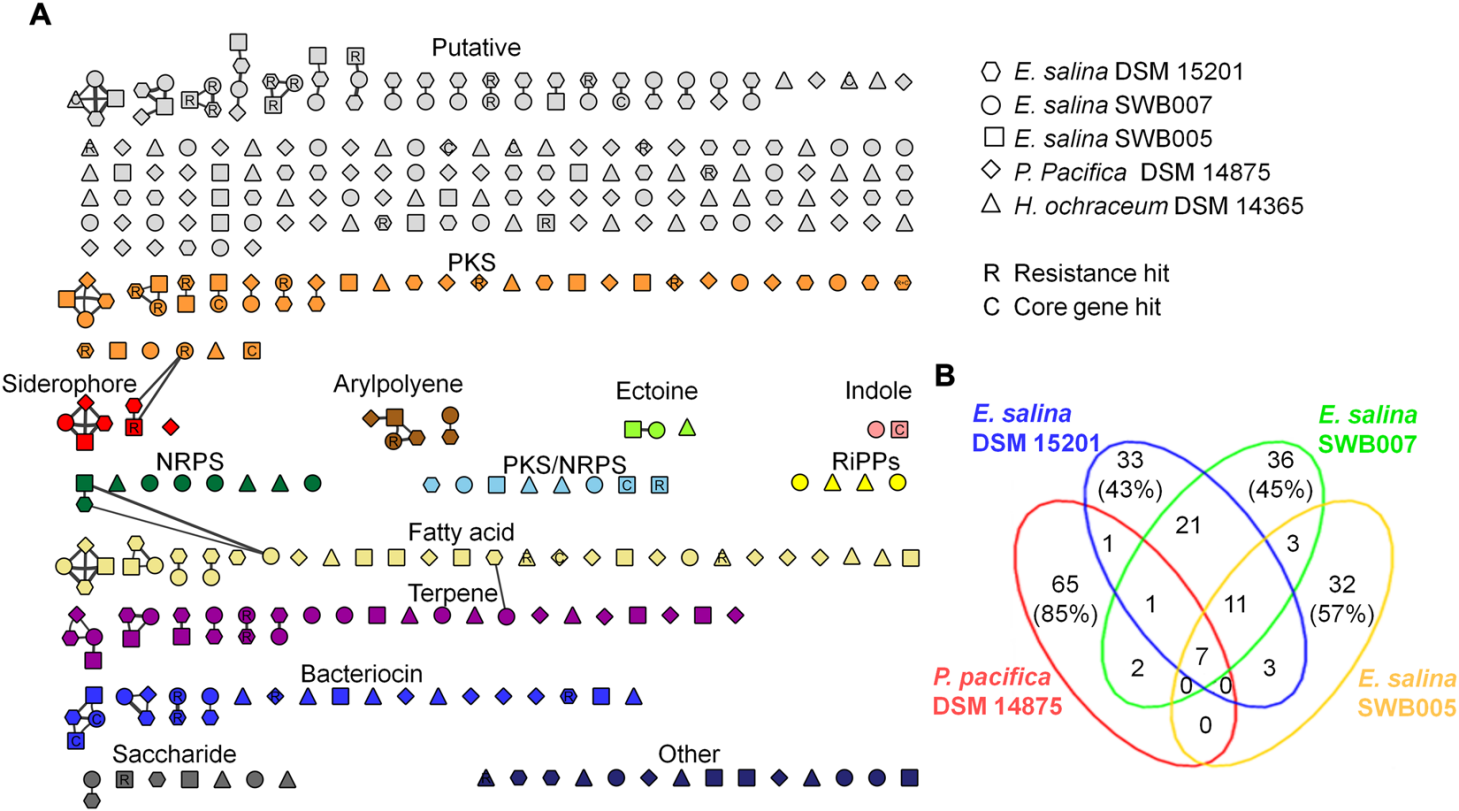
Similarity network of the predicted biosynthetic gene clusters (BGCs) in the five analyzed genomes. (**A**) Unique and shared similar BGCs (connected by a line). ARTS hits for resistance (R) and essential core genes (C) are labeled inside the respective nodes. (**B**) Venn diagram displaying node counts according to distribution in strains (*H. ochraceum* is excluded, since this strain has only 1 BGC which is similar to a BGC of the other strains). Interactive network is available at http://www.ndexbio.org under the title (Fig. 5) or by the DOI (http://doi.org/10.18119/N9F30V).

To get additional insights into the nature of the metabolites putatively corresponding to a BGC, an analysis using the ARTS webserver was performed^35^. This tool aims to enable prioritization of BGC, which correspond to antibacterial compounds. It is based on the fact that, to avoid suicide, an antibiotic producer harbors resistance genes often within the same BGC responsible for manufacturing of the compound. Known resistance, as well as possible resistant housekeeping genes are detected^35^. Using this analysis, several resistance model hits were identified (Table 2 and Fig. 5) suggesting that these specific BGCs code for antibacterial compounds. 7 to 13 resistance model hits were identified among the *E*. *salina* strains, including beta-lactamase, ABC-transporters, and other efflux systems. In *P*. *pacifica* DSM 14875 and *H*. *ochraceum* DSM 14365 only 4 hits pointing toward antibacterials were identified.

In a next step, we analyzed if BGCs encoding specialized metabolites are shared between the myxobacterial strains. A similarity network of all detected BGCs in the genomes was created based on the Pfam similarity metrics^34^. Out of the 351 BGCs identified, 124 (35%) can be found in at least two strains (Fig. 5A). The closely related strains *E*. *salina* SWB007 and *E*. *salina* DSM 15201 have the biggest overlap, whereby more than two third (71%) of the BGCs show similarities (Table S1A). *E*. *salina* SWB005 and *P*. *pacifica* DSM 14875 share several similar gene clusters with at least one other strain in the network (19.3% and 8.9%, respectively). Conversely, *H*. *ochraceum* DSM 14365 has only one BGC in common with other strains. This BGC is annotated as putatively related to 3-hydroxybutyryl-CoA biosynthesis. In fact, excluding *H*. *ochraceum*, only seven BGCs equivalent to 9–11% of the BGCs in each strain are similar between all *E*. *salina* strains and *P*. *pacifica* (Fig. 5B). However, 38.7% of the shared similar BGCs are categorized as putative, meaning that no corresponding metabolite class can be predicted (Table S1B). From the predictable BGCs, PKS clusters contribute to the highest share with 14.5%, followed by terpene (12.1%), and fatty acid (11.3%) BGCs (Table S1B). If only the strain specific (unique) BGCs are considered, half of them (50.7%) are classified as putative. The other half of the unique BGCs can be linked to the biosynthesis of polyketides (9.7%), fatty acids (8.8%), others (6.1%), and further less abundant ones (Fig. 5A and Table S1B). BGCs coding for peptidic metabolites, e.g. encoding NRPSs, PKSs/NRPSs and RiPPs, are mostly strain specific in the investigated strains.

In a next step, the predicted biosynthetic pathways were analyzed in more detail.

#### Terpenes

Many of the shared specialized metabolite BGCs encode for the biosynthesis of terpenes. The *E*. *salina* strains harbor six to nine terpene BGCs, *P*. *pacifica* DSM 14875 five and *H*. *ochraceum* DSM 14365 only three. *In silico* metabolic analysis using RAST revealed that all strains harbor the potential to generate the building blocks necessary for terpene assembly (Figs S6–S8). Several of the identified terpene BGCs could be linked to known terpene BGCs, including geosmin, squalene, sterols and carotenoids.

The predicted geosmin BGC shows high similarity to the BGC of *Nostoc punctiforme* PCC 73102 (ATCC 29133), which was investigated before^42^. Beside the gene encoding the geosmin synthase/cyclase, two genes encoding transcription regulators were also detected (Fig. S9). All strains except *P*. *pacifica* DSM 14875 harbor this cluster. The same gene cluster can be also found in the closely related halophilic myxobacterium *Nannocystis exedens* ATCC 25963 (Fig. S9). Interestingly, in this bacterium, 2-methylisoborneol and geosmin were identified as the main volatile compounds^43^. A squalene BGC was detected in all five investigated strains. This BGC encodes two squalene synthases (HpnC and D) and a squalene-associated FAD-dependent desaturase (HpnE), necessary to convert farnesyl diphosphate (FPP) to squalene (Fig. S10). In addition, the *E*. *salina* strains harbor three conserved squalene/hopene cyclases in other locations of their genomes, while *P*. *pacifica* DSM 14875 harbors two. The squalene/hopene cyclases detected in one of the BGC conserved in all *E*. *salina* strains and *P*. *pacifica* DSM 14875 showed BLAST hits towards different described sterol synthases including lanosterol and cycloartenol synthases (Fig. S11). *H*. *ochraceum* DSM 14365 does not harbor any additional squalene/hopene cyclase. Furthermore, a carotenoid BGC was found to be shared between all investigated strains. The essential genes for geranylgeranyl-CoA diphosphate synthase, a phytoene synthase, two dehydrogenases and a polyprenyltransferase are present^44^ (Fig. S12).

#### Polyketides (PKs)

The biggest group of specialized metabolite BGCs is linked to polyketides, *i*.*e*. 11.4% of all BGCs (Fig. 5). The total count of polyketide BGCs is 9–11 for *E*. *salina* strains and *P*. *pacifica* DSM 14875, while *H*. *ochraceum* DSM 14365 harbors only two. The genes coding for biosynthesis of starter and extender units for polyketide assembly were identified (see SI for details). Beside the standard extender unit malonyl-CoA (mCoA), the results indicate that the strains also possess the potential to synthesize methylmalonyl-CoA (mmCoA) and propionyl-CoA (pCoA). The latter is formed in the catabolism of isoleucine and valine (Fig. S7) and can serve as precursor for mmCoA. Ethylmalonyl-CoA (emCoA) can be biosynthesized through carboxylation of butyryl-CoA (bCoA). Carboxylation of bCoA is a described side activity of the propionyl-CoA carboxylase (PCC), which is part of the mmCoA biosynthesis (see above). Another pathway yielding emCoA is the conversion of crotonyl-CoA (cCoA) to emCoA by the catalytic activity of a cCoA carboxylase/reductase (CCR). A gene putatively coding for this conversion was identified in *E*. *salina* SWB007, *i*.*e*. annotated as crotonyl-CoA reductase /alcohol dehydrogenase (accession: WP_106090768), 61% identity to Leu10 and 51% identity to TgaD, which are part of leupyrrin and thuggacins BGCs in *Sorangium cellulosum*^45,46^. It is of interest that none of the polyketide BGCs in these bacteria could be linked to any known polyketide BGC and also they are just partly similar to BGCs of terrestrial myxobacteria and streptomycetes. For example, a putative type 1 PKS BGC is shared between *E*. *salina* strains and *P*. *pacifica* DSM 14875, shows some similarities to the thuggacin BGC from *Chondromyces crocatus*^45^ (Fig. S13). However, the corresponding metabolite to this BGC is unknown.

In addition, there are some type III polyketide synthase (PKSIII) BGCs found in analyzed strains except *Haliangium ochraceum* DSM 14365. *P*. *pacifica* DSM 14875 harbors one and *E*. *salina* DSM 15201, SWB007, and SWB005 harbor two, three and four PKSIII BGCs, respectively. One PKSIII BGC is shared between *E*. *salina* strains and *P*. *pacifica* DSM 14875, while another PKSIII BGC is shared only between the *E*. *salina* strains. Furthermore, *E*. *salina* SWB007 carries a unique PKSIII BGC, consisting of genes encoding a PKSIII, a methyltransferase, and an oxidoreductase. In its vicinity, genes encoding a polyprenyl synthetase and a polyprenyl transferase were detected (Fig. S14).

#### Non-Ribosomal Peptides (NRPs) and PKs/NRPs hybrids

Almost all of NRPS and PKS/NRPS hybrid BGCs were strain specific and only identified in *E*. *salina* strains and *H*. *ochraceum* DSM 14365. In *E*. *salina* SWB007, a strain specific type 1 PKS/NRPS BGC was identified, showing high homology to the reported leupyrrin BGC from *Sorangium cellulosum* So ce690^46^. In depth investigation of the gene cluster revealed that all genes necessary for leupyrrin biosynthesis are present (Fig. S15).

#### Bacteriocins

Several bacteriocin BGCs were identified in each strain. The similarity network (Fig. 5) indicated many of the them to be similar BGCs, i.e. 11 out of 19 have at least one counterpart, if the *E*. *salina* strains and *P*. *pacifica* DSM 14875 are considered.

#### Arylpolyenes

Arylpolyene (APE) BGCs were detected only in *E*. *salina* strains and *P*. *pacifica* DSM 14875. One of them is well conserved within all with homologous gene clusters from different marine photobacterium strains and closely resembles the APE BGC of *Escherichia coli* CFT073 and of *Vibrio fischeri* ES114 (100% of the biosynthetic genes show similarity, Fig. S16)^2^. Another APE BGC was only found in *E*. *salina* SWB007 and *E*. *salina* DSM 15201. However, the latter one did not show high similarity to any known BGCs.

#### Siderophores

Siderophore BGCs (NRPS-independent) were only shared between the *E*. *salina* strains and *P*. *pacifica* DSM 14875. Each strain harbors two distinct siderophore BGCs. One of them contains only one conserved gene from the IucA/IucC family of siderophore biosynthesis enzymes and the other encodes two IucA/IucC-like proteins and a lysine/ornithine N-monooxygenase.

#### Ectoine and hydroxyectoine

A complete ectoine/hydroxyectoine BGC was detected only in *E*. *salina* SWB005 and SWB007. In *H*. *ochraceum* DSM 14365 only an ectoine synthase gene was detected, while all the other necessary genes were absent. In addition, the ectoine BGC in *E*. *salina* SWB007 contains a glycine/sarcosine N-methyltransferase (GSMT) and a sarcosine/dimethylglycine N-methyltransferase (SDMT), which are responsible for betaine biosynthesis (Fig. S17)^47^.

#### Indole

All *E*. *salina* strains harbor a conserved indole prenyltransferase. However, the adjacent genes are either rearranged or not conserved (Fig. S18).

#### Ribosomally synthesized and post-translationally modified peptides (RiPPs)

BGCs coding for RiPPs were only found as unique BGCs in the genomes of *E*. *salina* SWB007 and *H*. *ochraceum* DSM 14365. A lanthipeptide and a thiopeptide BGC were detected in the genome of *E*. *salina* SWB007, and in *H*. *ochraceum* DSM 14365 a lanthipeptide and a lassopeptide BGC were detected.

#### Putative gene clusters

Many of the putative BGCs (29%) were shared as similar BGCs between *E*. *salina* strains and *P*. *pacifica* DSM 14875. They are mostly related to the biosynthesis of primary metabolites, such as a BGC putatively linked to the production of 3-crotonyl-CoA and 3-hydroxybutyryl-CoA. In addition, a conserved PHB synthase identified in *E*. *salina* strains and *P*. *pacifica* DSM 14875 are probably involved in the synthesis of polyhydroxybutyrate (PHB) from 3-hydroxybutyryl-CoA (Fig. S19).

### Metabolomic analysis of four marine myxobacterial strains

**Figure 6:**
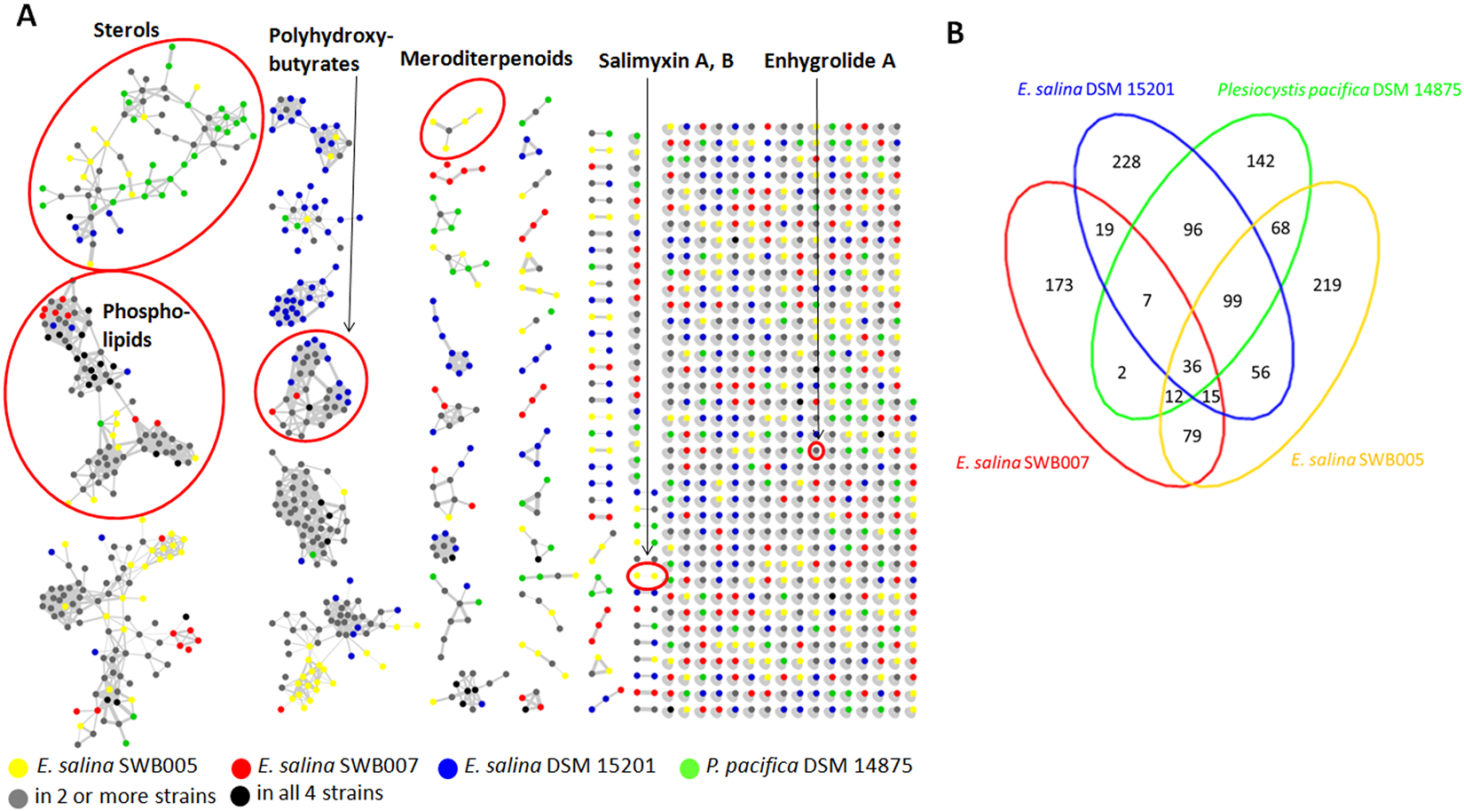
(**A**) Molecular network of *E. salina* SWB005, SWB007, DSM15201 and *P. pacifica* DSM 14875 extracts and fractions. Network is color-coded according to detection from single or multiple strains. Identified specialized metabolites are marked. (**B**) Venn diagram displaying node counts according to ion distribution in strains. Interactive network is available at http://www.ndexbio.org under the title (Fig. 6.) or by the DOI (http://doi.org/10.18119/N9988C).

Next, we aimed to analyze and compare the metabolomes of the marine myxobacterial strains, in order to see if the bioinformatics results are translatable into actual metabolites. For this type of analysis, the more closely related strains *E*. *salina* SWB005, SWB007, DSM15201 and *P*. *pacifica* DSM 14875 were selected. The strains were cultivated in liquid medium containing adsorber resin and subsequently extracted and fractionated. The crude extracts and all fractions were analyzed with HPLC coupled with high-resolution mass spectrometry and automated fragmentation (HPLC-HRMS/MS). The resulting MS^2^ data were used to generate a molecular network consisting of 1251 nodes after removal of media blanks (Fig. 6A). The ion distributions were counted and summarized in a Venn diagram (Fig. 6B). *E*. *salina* SWB005 and DSM15201 display the highest metabolic diversity of the four strains with 584 and 556 nodes, respectively, contributing to the network. Interestingly, all four strains show a relatively high percentage of strain-specific nodes, *i*.*e*. nodes that were only found in one strain. The most unique metabolome shows *E*. *salina* SWB007, where more than half of all nodes (173 of 343) were found to be strain-specific. Surprisingly, only 6–11% of the nodes in each strain were shared in the network by all four strains. Taken together, this analysis points to a large degree of unique metabolism in all four investigated strains under laboratory conditions.

Only a few nodes in the network could be dereplicated as specialized metabolites using the GNPS and our metabolite libraries (Table S3). Salimyxin A and salimyxin B were previously isolated from *E*. *salina*^15^. Both compounds were detected as strain specific metabolites of *E*. *salina* SWB005 in this analysis. Retention time and exact mass of all compounds correspond to an authentic standard. Enhygrolide A^15^, was found in extracts from *E*. *salina* SWB005 and SWB007. In order to extend the metabolomic results, the completely uninvestigated *E*. *salina* strain SWB006 was included to the investigation. Hence, this strain was fermented, extracted and its metabolomic profile analyzed. Also from this strain, enhygrolide A was identified (Fig. S20). Large scale cultivation of this strain was required to enable isolation of the compound for verification. By this approach, enhygrolide A was isolated and its structure confirmed by NMR spectroscopy (Figs S21 and 22).

One metabolite cluster from the network with a mass range between 883.3554-1332.5815 *m*/*z* displayed several characteristic mass shifts of 86.04 Da, which correspond to the loss or gain of hydroxybutyric acid (Fig. S23A). In addition, in the MS^2^ spectra of these compounds, several hydroxybutyric acid mass shifts were observed (Fig. S23B). Thus, we conclude that this metabolite cluster consists of different molecule weight fragments of the polymer polyhydroxybutyric acid (PHB), which is produced by all the strains. These biopolymers gained interest due to their biodegradability, biocompatibility, the possibility of biosynthesis from renewable resources, and similar physical and chemical characteristics to the ones of petrochemical polymers^48^. Other compound clusters in the network could be dereplicated with the help of the GNPS library search tool. These include a number of ions annotated as triterpenes/sterols and a large group of phospholipid-related molecules. Finally, with the DEREPLICATOR+ tool available on the GNPS platform^49^, one metabolite cluster produced by *E*. *salina* SWB005 and *P*. *pacifica* could be annotated with high confidence as meroditerpenoids related to tetraprenyltoluquinols isolated from marine algae^50^.

## Discussion

Obligate marine myxobacteria have been discovered only recently compared to their terrestrial counterparts. Since then, a small number of marine myxobacterial strains and specialized metabolites were isolated^51^. However, by metagenomics approaches, 16 S rRNA gene sequences of marine myxobacteria were identified from sediments of different locations, depths, and climatic regions, indicating that they are widely distributed around the globe. This suggests that the vast majority of marine myxobacteria has yet to be discovered. Furthermore, they are separated from terrestrial myxobacteria at high levels of classification^52,53^. This indicates a high chance for the discovery of novel chemical scaffolds, since recently a correlation between taxonomic distance and the production of distinct secondary metabolite families was proven^54^. Therefore, marine myxobacteria should be a bioresource for novel specialized metabolites because their terrestrial counterparts are one of the prime sources of these bioactive compounds^51,53^.

Similar to other marine Deltaproteobacteria, marine myxobacteria can be isolated from samples taken from benthic ecosystems such as sediments, sea weeds, sea grasses and aggregates close to the sediment surface^9,55,56^. However, to date the cultivation of marine Myxobacteria lags far behind to terrestrial ones. One main obstacle to their isolation is the slow growth with the consequence that marine myxobacteria are easily overgrown by other faster growing bacteria. Another problem is, that usually more than one cell is needed for these social bacteria to start growing on agar plates and they usually prefer media poor in nutrients^16^.

Here, we could show by comparative genomic analysis that the marine-derived species harbor an enormous potential for the discovery of novel natural products. The five available genomes of marine myxobacteria revealed that a relatively large portion of the genome (~10%) is dedicated to various classes of BGCs, corresponding to the production of specialized metabolites^12^.

The five marine myxobacteria from the family Nannocystaceae, for which genome information is available, are related to each other as evidenced by a conserved core genome. However, *in silico* parameters, *i*.*e*. ANI, isDDH, and difference of the GC content, clearly indicate that all *E*. *salina* strains investigated in this work should be classified as different species. In fact, it seems that significant parts of the genomes are either from different ancestral origin or have diverged rapidly. The same situation was observed in terrestrial myxobacteria, which show a large variation in their genomes and a small core genome^57,58^.

For the unique BGCs of the marine strains, the corresponding metabolites are so far unknown. However, the observation that BGCs related to PK and terpene biosynthesis represent the most abundant BGC types, is in line with the fact that most of the compounds isolated so far from myxobacteria, terrestrial or marine ones, are terpenoids, PKs, NRPs and PK/NRP hybrids^12,14,43,59–62^.

Myxobacteria, along with actinobacteria and cyanobacteria harbor the majority of the annotated terpene synthases among all bacteria^60^. Many terpenes are volatile compounds and might play a communication role during the multicellular life stages in myxobacteria^436^. Interestingly, conserved terpene BGCs of the marine strains can be attributed to different classes of terpenoids, e.g. carotenoids, sterols, and geosmin. The latter compound was thought to be indicative for terrestrial strains and was unexpected to be present in the genomes of the marine strains. Several sterols like lanosterol, cycloartenol and zymosterol were already reported from *E*. *salina* DSM15201 and *P*. *pacifica* DSM 14875^14^, and also the metabolomic analysis indicates a variety of sterols synthesized by these strains. The presence of the squalene BGC in all investigated strains emphasizes the importance of this compound as an intermediate in the biosynthesis of sterols, hopanoids, and related pentacyclic triterpenes with numerous essential functions, including the stabilization of lipid membranes and formation of membrane rafts^63^. It can be speculated that this represents important features for the adaptation to the marine environment, like the presence of BGCs for compatible solutes, e.g. ectoine and betaine^47^. Further, the carotenoid BGC in marine myxobacteria is similar to the well-known carotenoid gene cluster in *Myxococcus xanthus*, producing several different carotenoids, mainly phytoene, esterified carotenoids and all-trans-phytoene with different colors such as yellow, orange and red^62^. Such a finding could be expected, since the phenotypic appearance of the strains on solid as well as in liquid medium is yellowish to orange. The presence of several strain specific terpene BGCs contributes to the remarkable complexity and diversity of terpene metabolism in these bacteria.

Our analysis shows that PK BGCs are abundant and conserved in the analyzed genomes. Several resistance and essential core genes detected within the cluster boundaries indicate that the corresponding metabolites will have antibacterial properties. However, only few metabolites with a polyketide backbone, e.g. haliamide and haliangicin, have been isolated so far from marine myxobacteria. In addition, salimabromide might be, partly of polyketide origin. The structures of these metabolites suggest the incorporation of malonate, methylmalonate and ethylmalonate units^13,17,64^. Accordingly, the biosynthetic pathways for all these predicted polyketide extender units were identified. Beside the pathways for mCoA biosynthesis, also the genes coding for the biosynthesis of mmCoA and pCoA are conserved in all analyzed strains, whereby emCoA, the rare extender unit putatively used in salimabromide biosynthesis, can be generated from butyryl-CoA via the side activity of the PCC or from crotonyl-CoA via carboxylase activity of CCR. Both coding genes were identified in all analyzed genomes.

Unlike the polyketide BGCs, which are often shared between the strains, NRPS and PKS/NRPS hybrid BGCs are rare and mostly strain specific. *P*. *pacifica* DSM 14875 carries no NRPS BGC. Examples of PKS/NRPS hybrid BGCs from marine myxobacteria are very limited, *e*.*g*. haliamide from *H*. *ochraceum* DSM14365 and phenylnannolone A from the halotolerant myxobacterium *Nannocystis pusilla* B150 were described in detail^13,65^. Here, we identified a PKS/NRPS hybrid gene cluster encoding for leupyrrin in the genome of *E*. *salina* SWB007. Leupyrrin was isolated and its gene cluster was reported before from the terrestrial *Sorangium cellulosum* strain So ce690^46^. Comparison with this BGC reveals that all encoded proteins are homologues, showing over 50% amino acid identity and complete coverage. Only some rearrangements are observed overall.

Several bacteriocin BGCs were identified in the strains, shared as well as unique ones. It can be suggested that the marine strains use them to compete with other bacteria, since it was reported that *Myxococcus virescens* uses bacteriocins against *M*. *xanthus* as a competitive mechanism of territory establishment^66^. Further, it was speculated that specific bacteriocins contribute to the enrichment of species within myxobacterial fruiting bodies^67^. Fruiting body formation was also observed in these marine strains.

Additional genomic features, which might contribute to the adaptation to the marine environment, could be the capability for the biosynthesis of arylpolyenes and siderophores. The corresponding BGCs are widely distributed throughout Gram-negative bacteria^2^. Arylpolyenes are structurally and functionally similar to the well-known carotenoid pigments with respect to their polyene systems and protect bacteria against oxidative stress^68^. Siderophores, as iron scavengers, contribute to iron acquisition under low-iron conditions. Here, NRPS-independent siderophore BGCs were only identified in *E*. *salina* and *P*. *pacifica* strains, while *H*. *ochraceum* lacks these BGCs, like the terrestrial myxobacterium *M*. *xanthus*. It is reported that the presence of arylpolyene BGCs is changing within bacterial genera due to frequent BGC loss from the descendants of a cluster-harboring ancestor, and due to frequent horizontal gene transfer^2^. In the future, when more marine myxobacterial genomes will become available, it will be possible to judge which events took place. Within the here investigated strains the presence of the ectoine BGC was also specific for *E*. *salina* SWB005 and SWB007, while the other strains do not harbor this specific BGC. This might be due to different strategies to cope with salt stress. Our previous work revealed *E*. *salina* SWB007 biosynthesizing ectoine, hydroxyectoine and betaine at high salt concentration, while *P*. *pacifica* does not produce any specialized organic solutes and relies on amino acids accumulation as osmoprotective agents^47^.

The metabolomic analysis revealed a high diversity of chemical features between the investigated bacteria. Despite the differences, one chemical feature is shared in all analyzed strains, *i*.*e*. polyhydroxybutyric acid (PHB), which was identified by a large cluster of characteristic MS spectra (±86.04 Da) belonging to PHBs different in length. The biopolymer PHB plays an important role in long-term survival of bacteria under nutrient-scarce conditions by acting as carbon and energy reserve^69^. Additionally, polyhydroxyalkanoates enhance the stress tolerance of bacteria against transient environmental assaults such as ultraviolet (UV) irradiation, heat and osmotic shock^69^. For the fruiting body forming myxobacteria PHB might act as energy supply at nutrient limited conditions, and as protective agent for myxospores.

From the few compounds previously isolated from the marine strains, enhygrolide A was detected from *E*. *salina* SWB005 and is now also proven to be produced by other *E*. *salina* strains, i.e. SWB006 and SWB007. Instead, the salimyxins A and B were only detected as strain specific features of *E*. *salina* SWB005. These compounds are degraded sterols and could hypothetically be modified/degraded from lanosterol or other sterols in this strain^15^. However, such modifications of sterols in myxobacteria are still elusive^14^.

Overall, the percentage of chemical features (6–11%) shared between all analyzed strains is consistent with the small core genome of these bacteria (13–16% of each genome). In contrast, 30–50% of the chemical features are unique in single strains which is consistent with 21–40% of the singleton genes and 43–85% of strain specific BGCs. A similar trend was also revealed in a study of 13 *Pseudoalteromonas luteoviolacea* isolates. Only 2% of the metabolomics features and 7% of biosynthetic genes were shared between all strains, while 30% of all chemical features and 24% of the genes were unique to single strains^5^. Similarly, significant differences have been found in the specialized metabolomes of *M*. *xanthus* isolates from different locations^70^ and also in the marine actinomycete *Salinispora*, where 75 strains were analyzed and compared^71^. In conclusion, each of the investigated marine myxobacterial strains harbors a high unique genetic and metabolic diversity, rendering this group of microorganisms a promising source for novel specialized metabolites and predicting further diversity for future isolates.

However, the number of isolated compounds to date from these strains is much lower than this predicted potential. This can be mostly contributed to the fact that marine myxobacteria are hard to isolate and cultivate due to their slow growth and difficult handling. Thus, improved cultivation techniques for these bacteria must be developed in the future^72^ and optimal conditions for specialized metabolite production evaluated. Heterologous expression approaches of orphan gene clusters should be considered as an alternative strategy to tap the specific metabolome of these organisms. Molecular biological tools for such approaches are available and are undergoing a steady process of improvement^73^.

Combination of genomic and metabolomic analyses reveals the strain specific potential for specialized metabolite production, and which compounds are indeed accessible under given *in vitro* conditions. These are important data in the early stage of natural product discovery to select and prioritize strains and cultivation conditions.

## Electronic supplementary material

Supplementary Information
