# Supplementary material for: Analysis of the Genome and Metabolome of Marine Myxobacteria Reveals High Potential for Biosynthesis of Novel Specialized Metabolites
Source: Sci Rep. 2018 Nov 9;8:16600. doi: 10.1038/s41598-018-34954-y (PMC6226438; doi:10.1038/s41598-018-34954-y)
Supplement: Supplementary file 1 — Supplementary Information [file 41598_2018_34954_MOESM1_ESM.docx]

**Supplementary information**

**Analysis of the Genome and Metabolome of Marine Myxobacteria Reveals High Potential for Biosynthesis of Novel Specialized Metabolites**

Jamshid Amiri Moghaddam,^a^ Max Crüsemann,^a^ Mohammad Alanjary,^b^ Henrik Harms,^c,d^ Antonio Dávila-Céspedes,^a^ Jochen Blom,^e^ Anja Poehlein,^f^ Nadine Ziemert,^b^ Gabriele M. König,^a,*^ Till F. Schäberle^c,d,g*^

Institute for Pharmaceutical Biology, University of Bonn, Bonn, Germany^a^; Department of Microbiology and Biotechnology, University of Tübingen, Tübingen, Germany^b^; German Center for Infection Research (DZIF) Partner Site Cologne/Bonn, Bonn, Germany^c^; Institute for Insect Biotechnology, Justus Liebig University Giessen, Giessen^d^; Bioinformatics and Systems Biology, Justus Liebig University Giessen, Giessen, Germany^e^; Department of Genomics and Applied Microbiology and Göttingen Genomics Laboratory, Georg-August-University Göttingen, Göttingen, Germany^f^; Department of Bioresources of the Fraunhofer Institute for Molecular Biology and Applied Ecology, Giessen, Germany^g^.

**
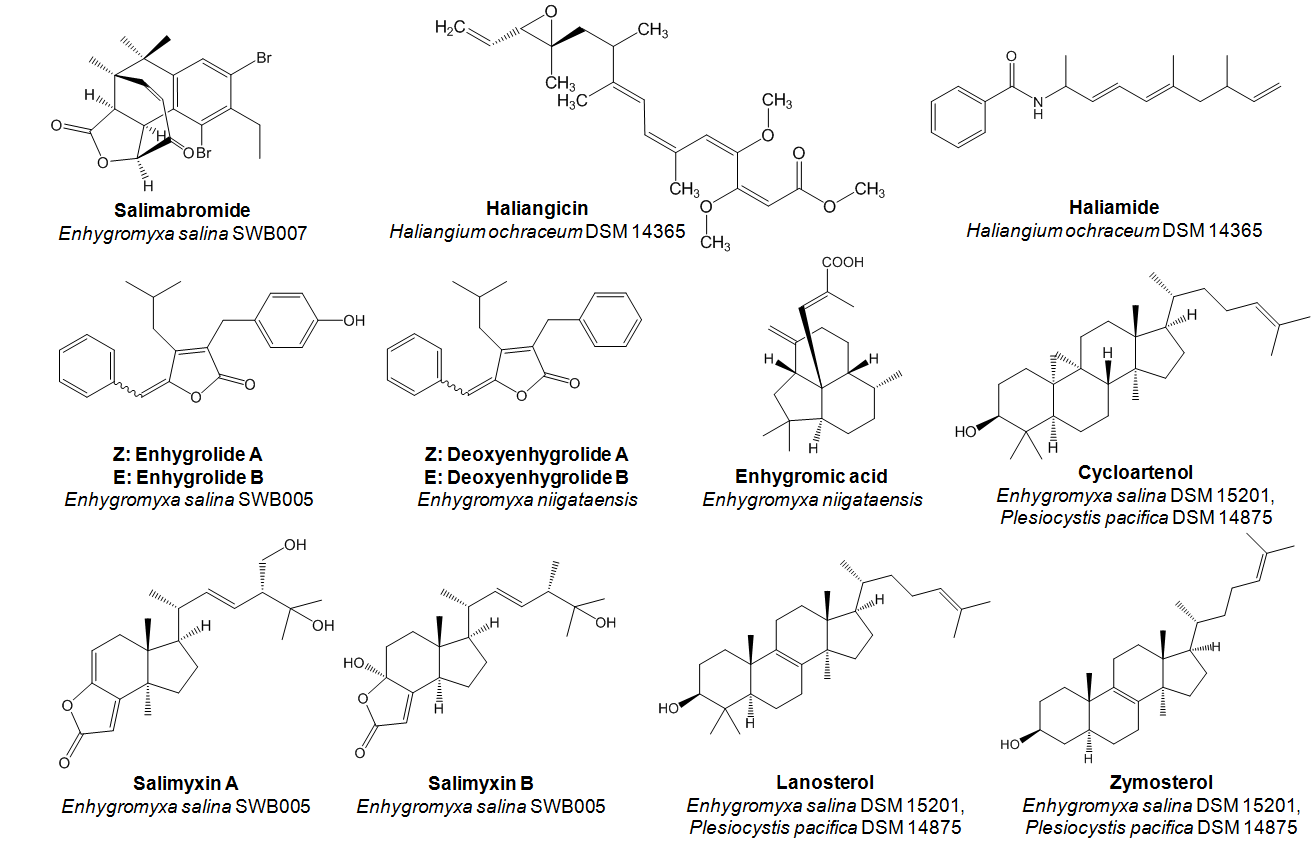
**

Figure S1. Structures of specialized metabolites isolated from marine myxobacteria

**
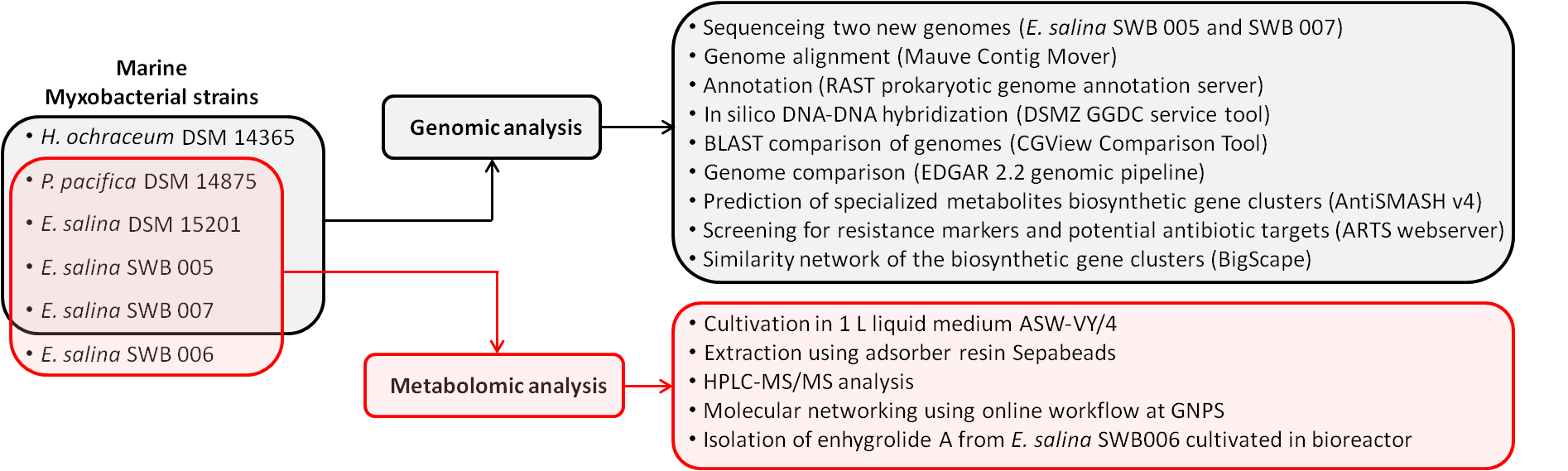
**

**Figure S2.** Schematic workflow of the study indicating which strains underwent which experiments.

**List of all the bioinformatics tools used to create the results for the manuscript with some general requirements:**

- **SPAdes** – St. Petersburg genome assembler – is an assembly toolkit containing various assembly pipelines. SPAdes version 3.10.1 was released under GPLv2 on March 1, 2017.
- **Trimmomatic** performs a variety of useful trimming tasks for illumina paired-end and single ended data. The selection of trimming steps and their associated parameters are supplied on the command line.
- **CheckM** provides a set of tools for assessing the quality of genomes recovered from isolates, single cells, or metagenomes. It provides robust estimates of genome completeness and contamination by using collocated sets of genes that are ubiquitous and single-copy within a phylogenetic lineage.
- **QUAST** stands for Quality Assessment Tool. The tool evaluates genome assemblies by computing various metrics. QUAST can evaluate assemblies both with a reference genome, as well as without a reference.
- **Mauve Contig Mover (MCM)** can be used to order a draft genome relative to a related reference genome. The MCM can ease a comparative study between draft and reference sequences by ordering draft contigs according to the reference genome.
- **Rapid Annotation using Subsystem Technology (RAST)** is a fully-automated service for annotating complete or nearly complete bacterial and archaeal genomes (http://rast.nmpdr.org/). It provides high quality genome annotations for these genomes across the whole phylogenetic tree.
- **EDGAR** is designed to automatically perform genome comparisons in a high throughput approach. EDGAR provides novel analysis features and greatly simplifies the comparative analysis of related genomes. Visualization features, like synteny plots or Venn diagrams, are offered to the scientific community through a web-based and thus platform independent user interface where the precomputed data sets can be browsed.
- **GGDC web service** reports digital DDH for a universal and accurate delineation of prokaryotic (sub-)species without inheriting the pitfalls of classic DDH, and also calculates differences in genomic G+C content.
- **CGView Comparison Tool (CCT)** is a package for visually comparing bacterial, plasmid, chloroplast, or mitochondrial sequences of interest to existing genomes or sequence collections.
- **AntiSMASH** (antibiotics & Secondary Metabolite Analysis Shell) is a free online tool that allows the rapid genome-wide identification, annotation and analysis of secondary metabolite biosynthesis gene clusters in bacterial and fungal genomes (http://antismash.secondarymetabolites.org/). It integrates and cross-links with a large number of in silico secondary metabolite analysis tools that have been published earlier. AntiSMASH is powered by several open source tools: NCBI BLAST+, HMMer 3, Muscle 3, Glimmer 3, FastTree, TreeGraph 2, Indigo-depict, PySVG and JQuery SVG.
- **Antibiotic Resistant Target Seeker (ARTS)** is provided by Ziemert lab in Tübingen and explores secondary metabolite gene clusters detected by AntiSMASH and uncover new resistant targets. It also screens secondary metabolite gene clusters for known targets, find unique metabolism and resistance factors in gene clusters and discover leads to novel antibiotic targets.
- **Biosynthetic Genes Similarity Clustering and Prospecting Engine (BiG-SCAPE)**. This program defines a distance metric between gene clusters using a combination of three indices (Jaccard Index of domain types, Domain Sequence Similarity the Adjacency Index).
- **Global Natural Products Social Molecular Networking (GNPS)** web-platform provides public data set deposition and/or retrieval through the Mass Spectrometry Interactive Virtual Environment (MassIVE) data repository. The GNPS analysis infrastructure further enables online dereplication, automated molecular networking analysis, and crowdsourced MS/MS spectrum curation.
- **Circos table viewer** turn data tables into chord diagrams and visualize them as circular figures. Through the settings panel it can be controled the manner in which the tabular data is parsed, filtered and displayed. E.g. display options used for figure 4: 1- Row/column segments in descending order. 2- Row segments placed first. 3- Row ribbons placed first. 4- Segment color is interpolated by segment count. 5- Ribbon color source is set to column. 6- Ribbon transparency set to 1/5. 7- Ribbon fade transparency set to 0/5. 8- Larger ribbons on top.
- **Cytoscape** (version 3.6.1) is an open source software platform for visualizing complex networks and integrating these with any type of attribute data. Cytoscape supports a lot of standard network and annotation file formats including GML. A variety of layout algorithms are available, including cyclic, tree, force-directed, edge-weight or manual Layouts. Networks display can be customized with different visual styles including node color, node shape, label, border thickness, or border color, and etc.

**
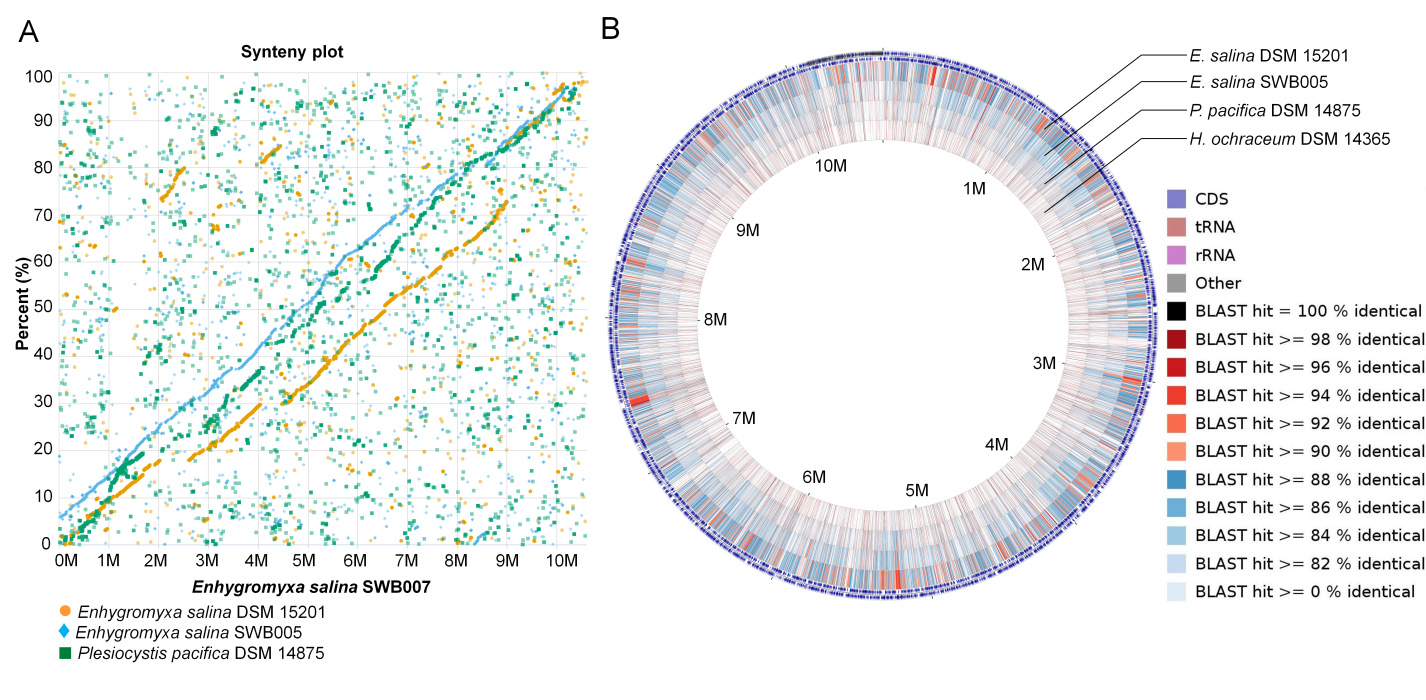
**

Figure S3. A: Synteny plot of selected genomes of marine myxobacteria within the contiguous contigs. *E. salina* SWB007 serves as reference. Positions within the reference genome are depicted on the x-axis, while the y-axis shows the relative position within the other genomes. Dots reflect the stop position of orthologous genes within the areas that reside inside contig boundaries. B: BLAST comparison of selected strains. *E. salina* SWB007 genome serves as reference and *E. salina* DSM 15201, *E. salina* SWB005, *P. pacifica* DSM 14875 and *H. ochraceum* DSM 14365 are indicated each by one ring.

**
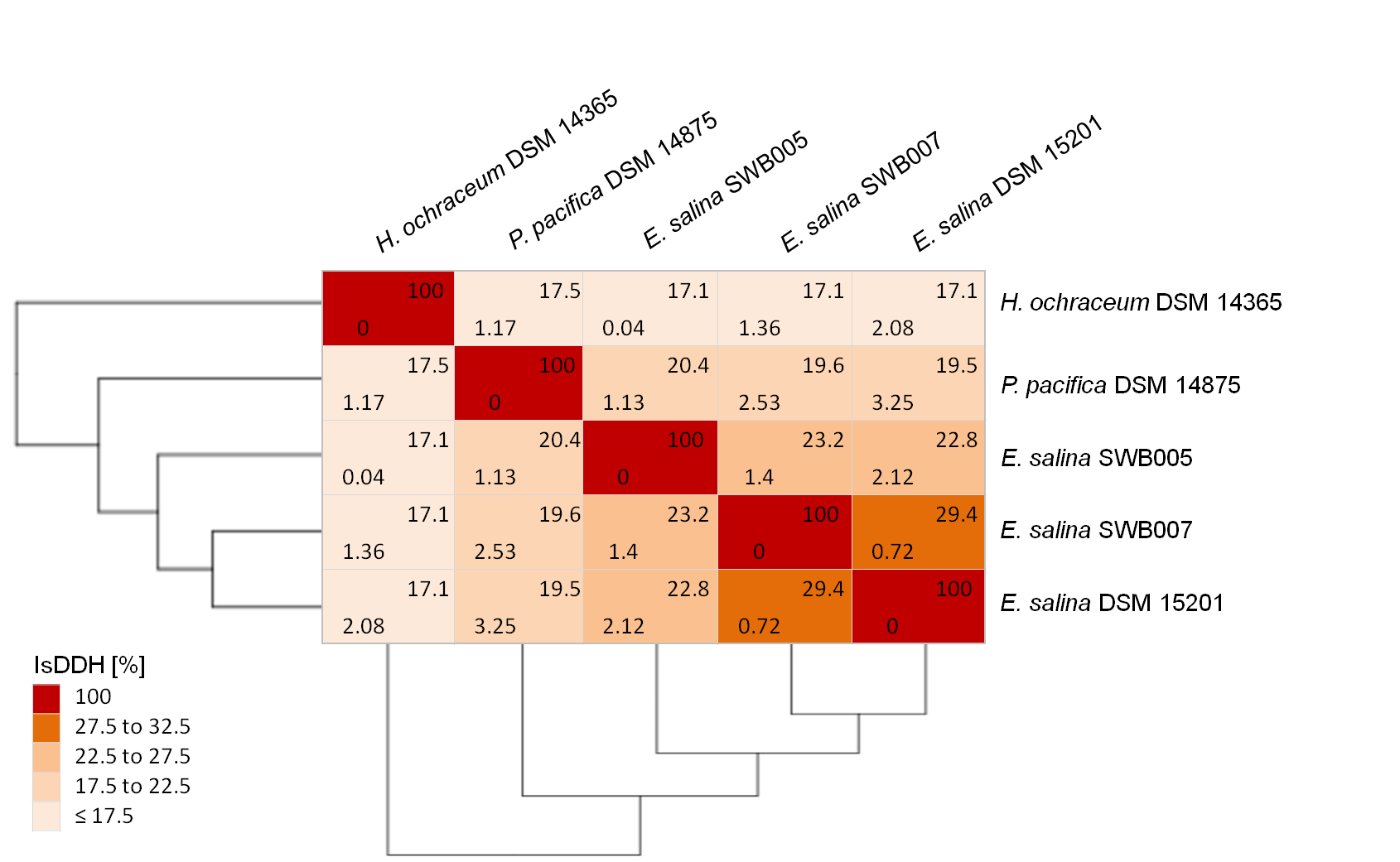
**

Figure S4. In silico DNA-DNA hybridization (isDDH) and difference in G+C content of the available marine myxobacteria genomes. The values on up-right side of each box represent isDDH and the values on down-left side are difference in G+C content. All values are given in percent.


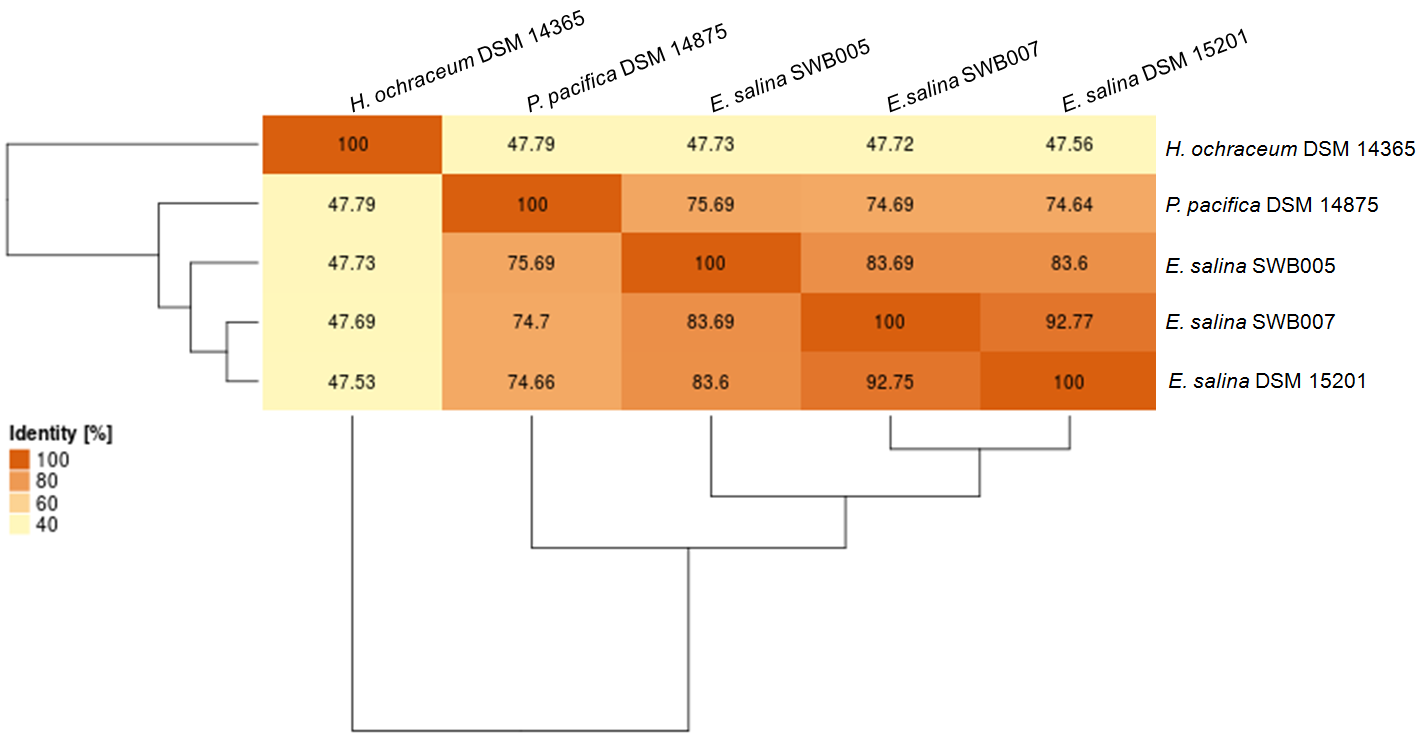


Figure S5. Average Amino Acids Identity (AAI) of the available marine myxobacteria. Orthologous genes of the core genomes were analyzed for their median identity.

Table S1. Contribution of the unique and shared similar BGCs in the similarity network (Figure 7A) based on strain (A) and cluster family (B).

| **A: Strain** | **Shared** | **(%)** |  | **Unique** | **(%)** |
| --- | --- | --- | --- | --- | --- |
| *E. salina* DSM 15201 | 44 | 35.5 |  | 33 | 14.5 |
| *E. salina* SWB007 | 44 | 35.5 |  | 36 | 15.9 |
| *E. salina* SWB005 | 24 | 19.3 |  | 32 | 14.1 |
| *P. Pacifica* DSM 14875 | 11 | 8.9 |  | 65 | 28.6 |
| *H. ochraceum* DSM 14365 | 1 | 0.8 |  | 61 | 26.9 |
| **Total** | **124** | **100** |  | **227** | **100** |
|  |  |  |  |  |  |
| **B: Cluster family** | **Shared** | **(%)** |  | **Unique** | **(%)** |
| Putative | 48 | 38.7 |  | 115 | 50.7 |
| PKS | 18 | 14.5 |  | 22 | 9.7 |
| Terpene | 15 | 12.1 |  | 13 | 5.7 |
| Fatty acid | 14 | 11.3 |  | 20 | 8.8 |
| Bacteriocin | 11 | 8.9 |  | 13 | 5.7 |
| Other | 0 | 0 |  | 14 | 6.1 |
| NRPS | 2 | 1.6 |  | 7 | 3.1 |
| Saccharide | 2 | 1.6 |  | 6 | 2.7 |
| Siderophore | 6 | 4.8 |  | 1 | 0.4 |
| PKS/NRPS | 0 | 0 |  | 9 | 4 |
| Arylpolyene | 6 | 4.9 |  | 0 | 0 |
| RiPPs | 0 | 0 |  | 4 | 1.8 |
| Indole | 0 | 0 |  | 2 | 0.9 |
| Ectoine | 2 | 1.6 |  | 1 | 0.4 |
| **Total** | **124** | **100** |  | **227** | **100** |

**Potential to generate the building blocks necessary for terpene assembly**

Terpene assembly can be achieved either through the mevalonate pathway, or by degradation pathways, *i.e.* of valine, leucine, isoleucine and the acyclic terpene degradation pathway (Fig. S6, S7 and S8). The precursors isopentenyl-PP, geranyl-PP, farnesyl-PP and geranylgeranyl-PP are expected to arise from the mevalonate pathway (Fig. S6). By degradation of valine, leucine and isoleucine, isovaleryl coenzyme A (3-methylbutanoyl-CoA), 3-hydroxy-3-methylglutaryl-CoA, acetyl-CoA, acetoacetyl-CoA and methylmalonyl-CoA can be built (Fig. S7). In addition, a putative BGC linked to the acyclic terpene utilization pathway (atu) can contribute acetate, acetyl-CoA and 7-methyl-3-oxo-6-octenoyl-CoA, whereby the latter yields 3-methylcrotonyl-CoA after two steps of β-oxidation (Fig. S8).


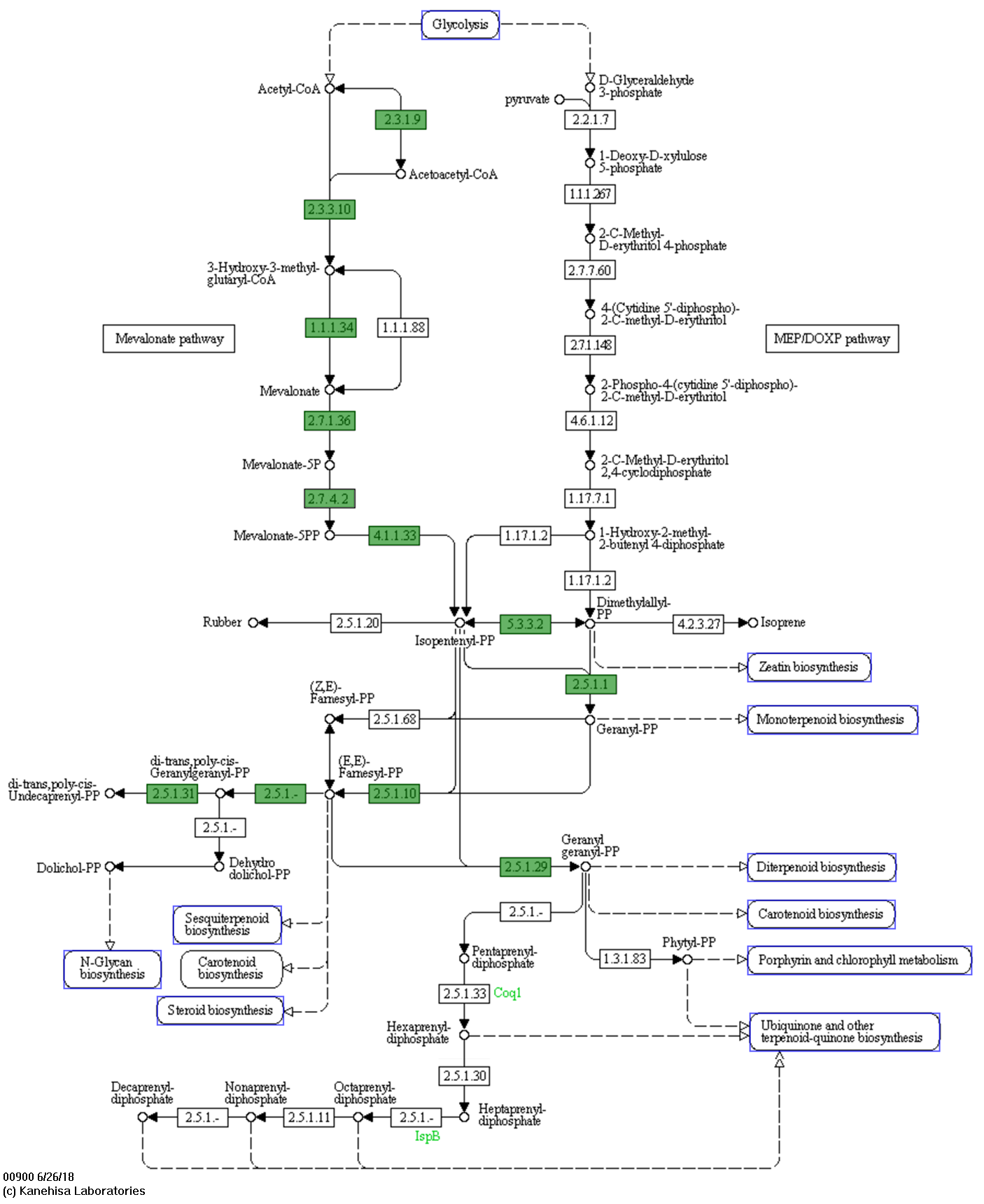


Figure S6. Terpenoid backbone biosynthesis pathway in *E. salina* SWB007. Green boxes indicate the presence of at least one feature for the respective reaction.


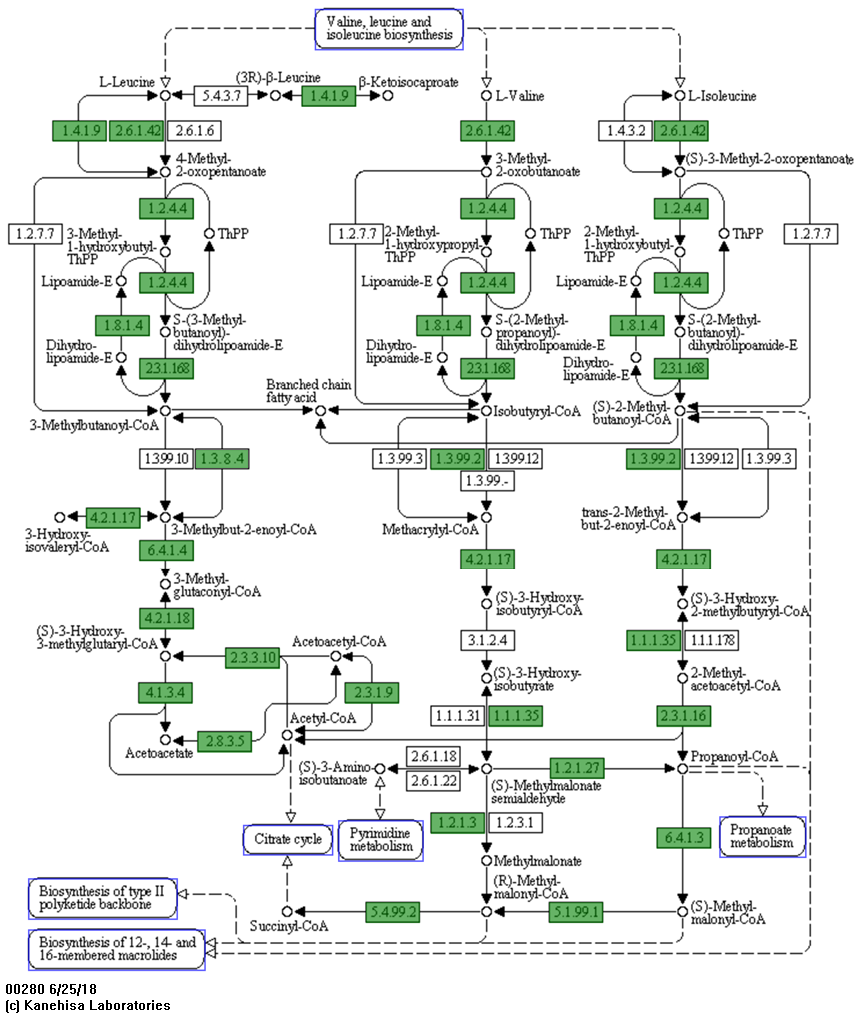


Figure S7. Leucine, isoleucine and valine degradation pathway of *E. salina* SWB007. Green boxes indicate the presence of the necessary encoding gene. The gene coding for EC 1.3.8.4 was identified manually by performing a BLAST search on the genome.


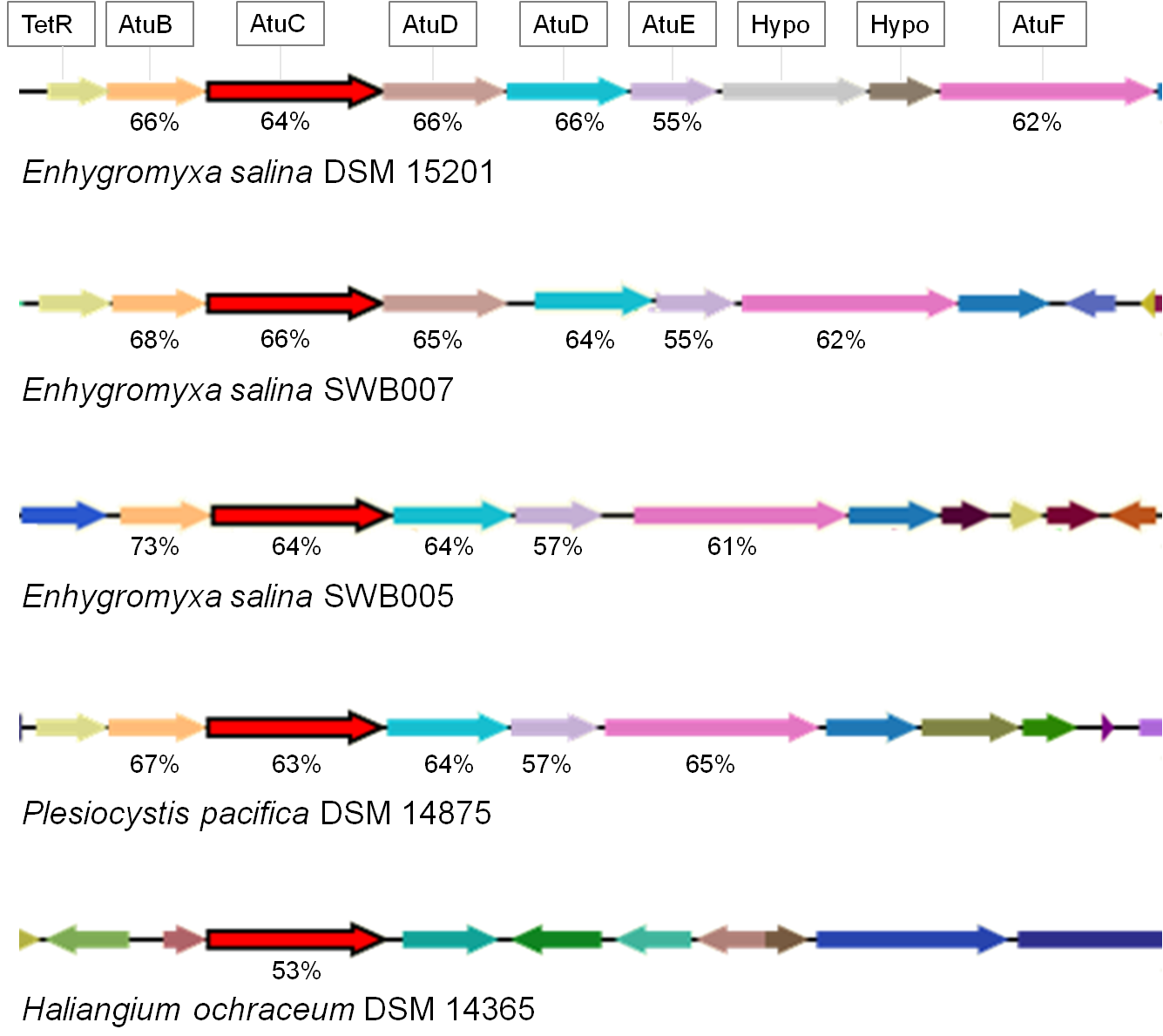


Figure S8. Acyclic terpene utilization (*atu*) gene cluster in the available genomes of marine myxobacteria. Identities (protein level) towards *Pseudomonas citronellolis* are given below the genes. The proteins encoded by the respective gene are indicated in the black boxes. Similar genes have the same color code. Hypo = Hypothetical protein.


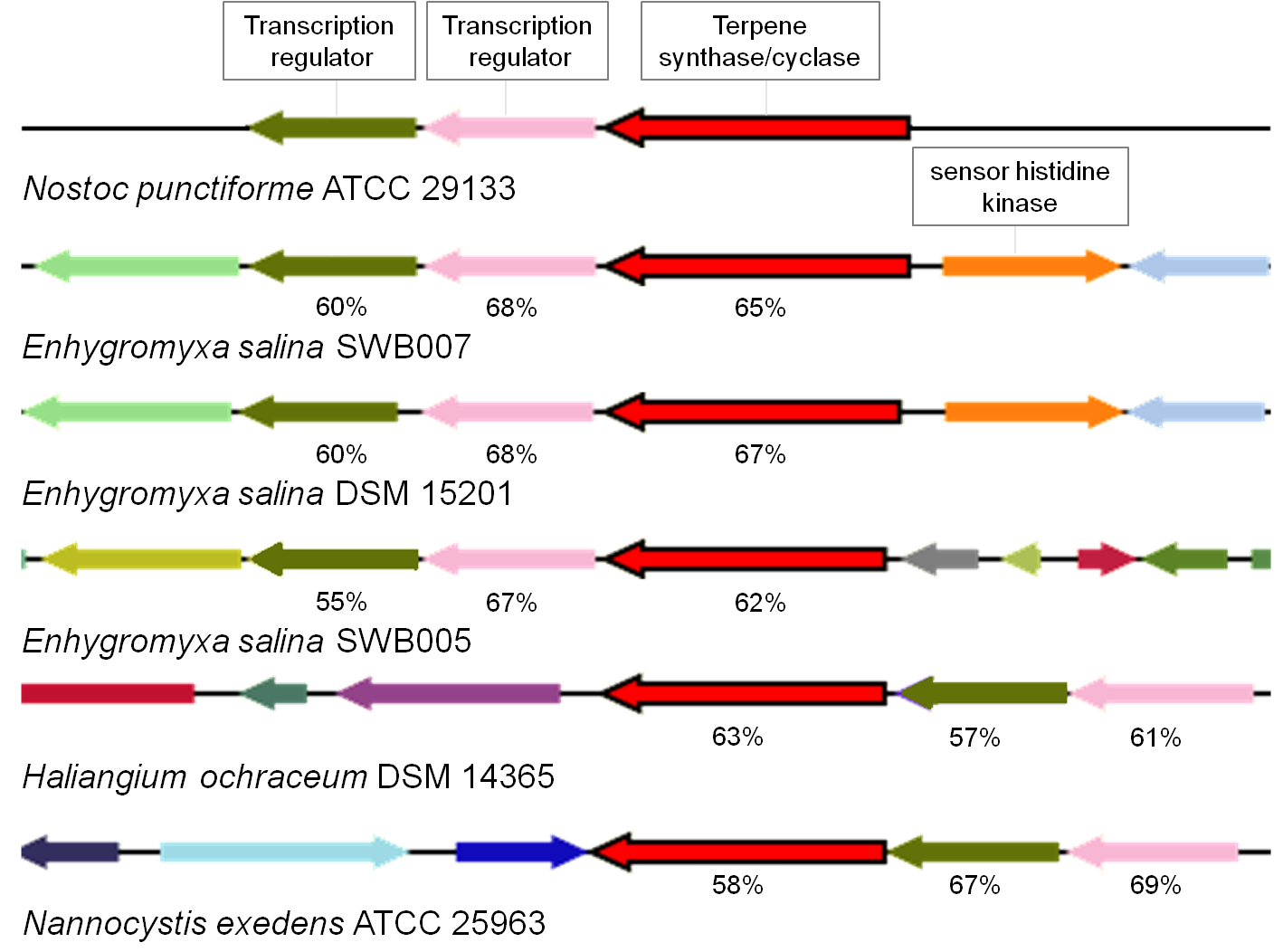


Figure S9. Geosmin BGC in available genomes of marine myxobacteria as well as *Nannocystis exedens* ATCC 25963. Identities of the genes products to the geosmin gene products of *Nostoc punctiforme* ATCC 29133 are given below. The same genes have similar color code.


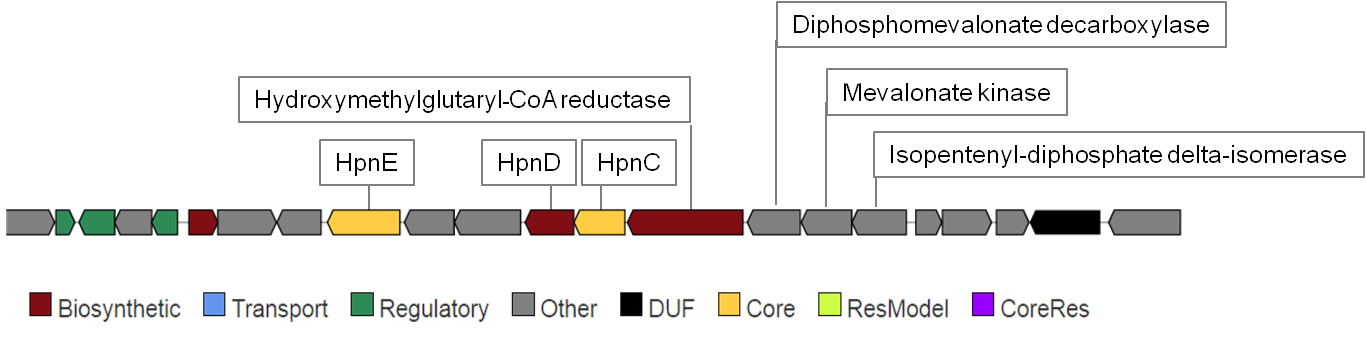


Figure S10. Biosynthetic gene cluster (BGC) for squalene biosynthesis in *E. salina* SWB007. The same BGC is present in all five available genomes of marine myxobacteria.


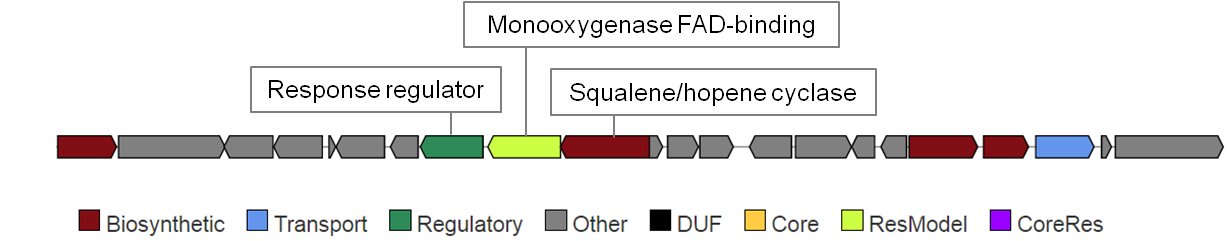


Figure S11. Conserved gene cluster for sterol biosynthesis in *E. salina* strains and *P. pacifica* DSM 14875. The genes of *E. salina* SWB007 are shown here as model.


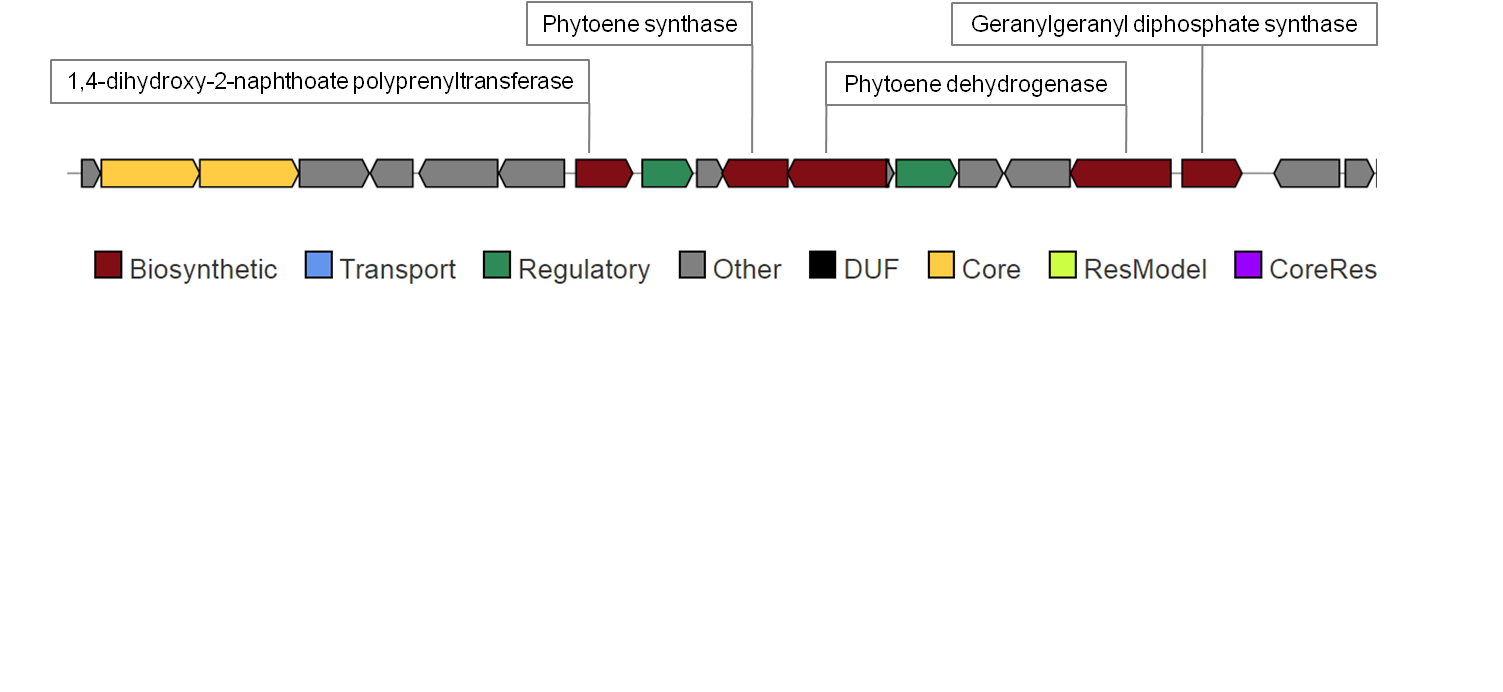


Figure S12. Conserved gene cluster for carotenoid biosynthesis in available genomes of marine myxobacteria. The genes of *E. salina* SWB007 are shown here as model.

**Biosynthesis of polyketides extender units**

Biosynthesis of extender units for polyketides biosynthesis were investigated using *in silico* analysis of the genomes. Conserved regions within the genomes of all strains revealed genes coding for the biosynthesis of malonyl-CoA. Therefore, four separate proteins are encoded: 1) Biotin carboxyl carrier protein of acetyl-CoA carboxylase (BCCP), 2) Biotin carboxylase of acetyl-CoA carboxylase (BC), 3) Acetyl-coenzyme A carboxyl transferase (CT) alpha chain and 4) Acetyl-coenzyme A carboxyl transferase (CT) beta chain.

As mentioned in terpene biosynthesis part, methylmalonyl-CoA (mmCoA) can be derived from propionyl-CoA (pCoA), which itself is formed in the catabolism of isoleucine and valine (Fig. S3). Carboxylation of pCoA to mmCoA can by catalyzed by a CT, which is present in all genomes analyzed. (Annotated as acetyl-coenzyme A carboxyl transferase alpha chain (EC 6.4.1.2)/acetyl-coenzyme A carboxyl transferase beta chain (EC 6.4.1.2)/propionyl-CoA carboxylase (PCC) beta chain (EC 6.4.1.3)).


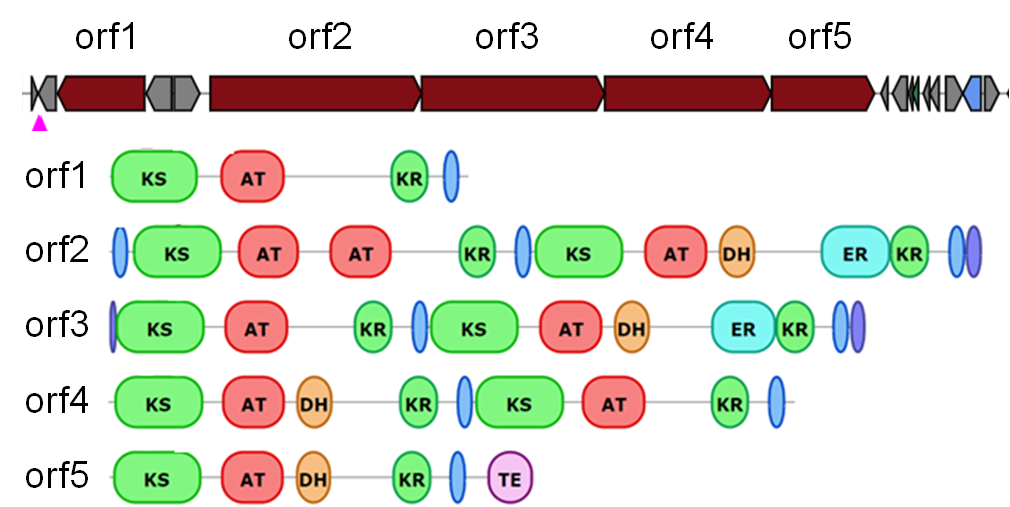


Figure S13. Type I PKS gene cluster found in *E. salina* strains and *P.pacifica* DSM14875. The genes of *E. salina* DSM 15201 are shown here as model.


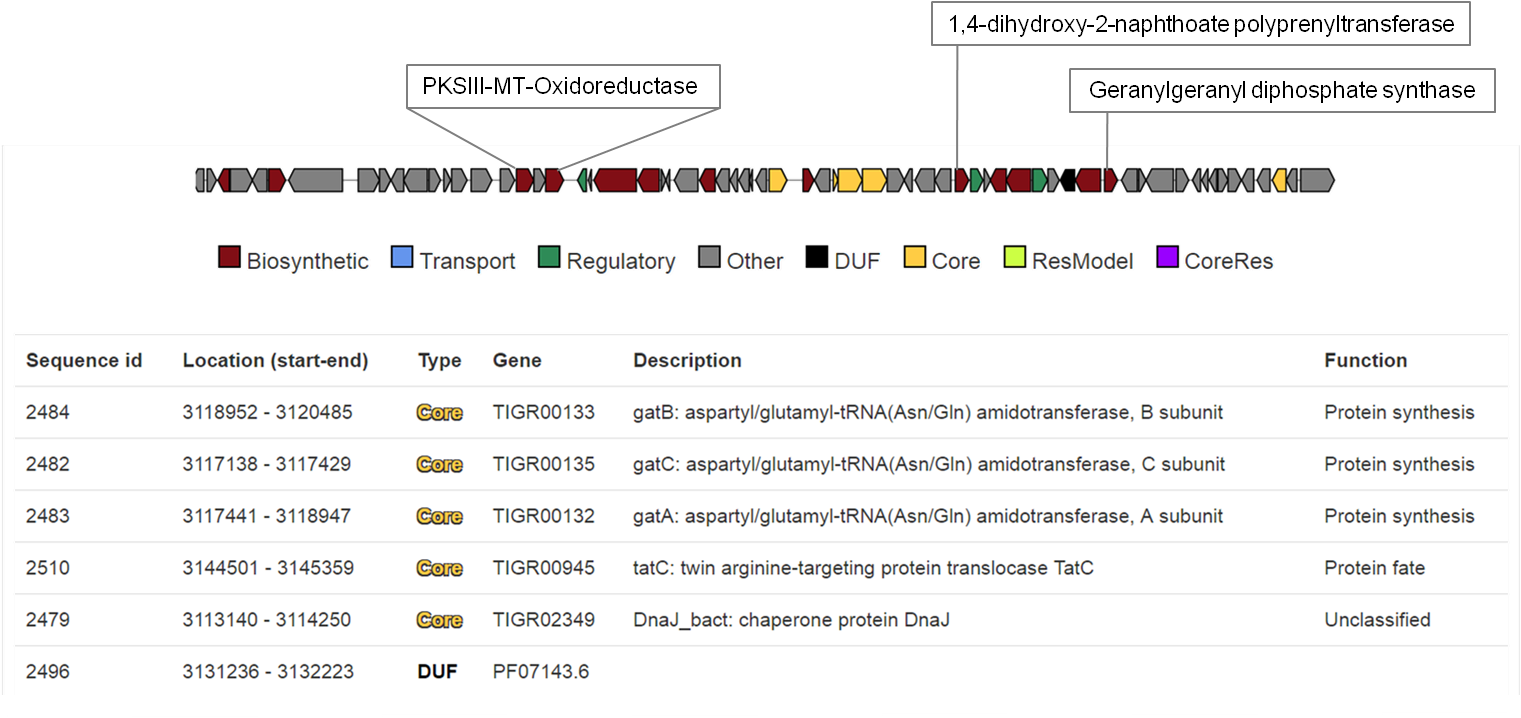


Figure S14. PKSIII BGC with genes associated with terpene biosynthesis in close proximity in *E. salina* SWB007.


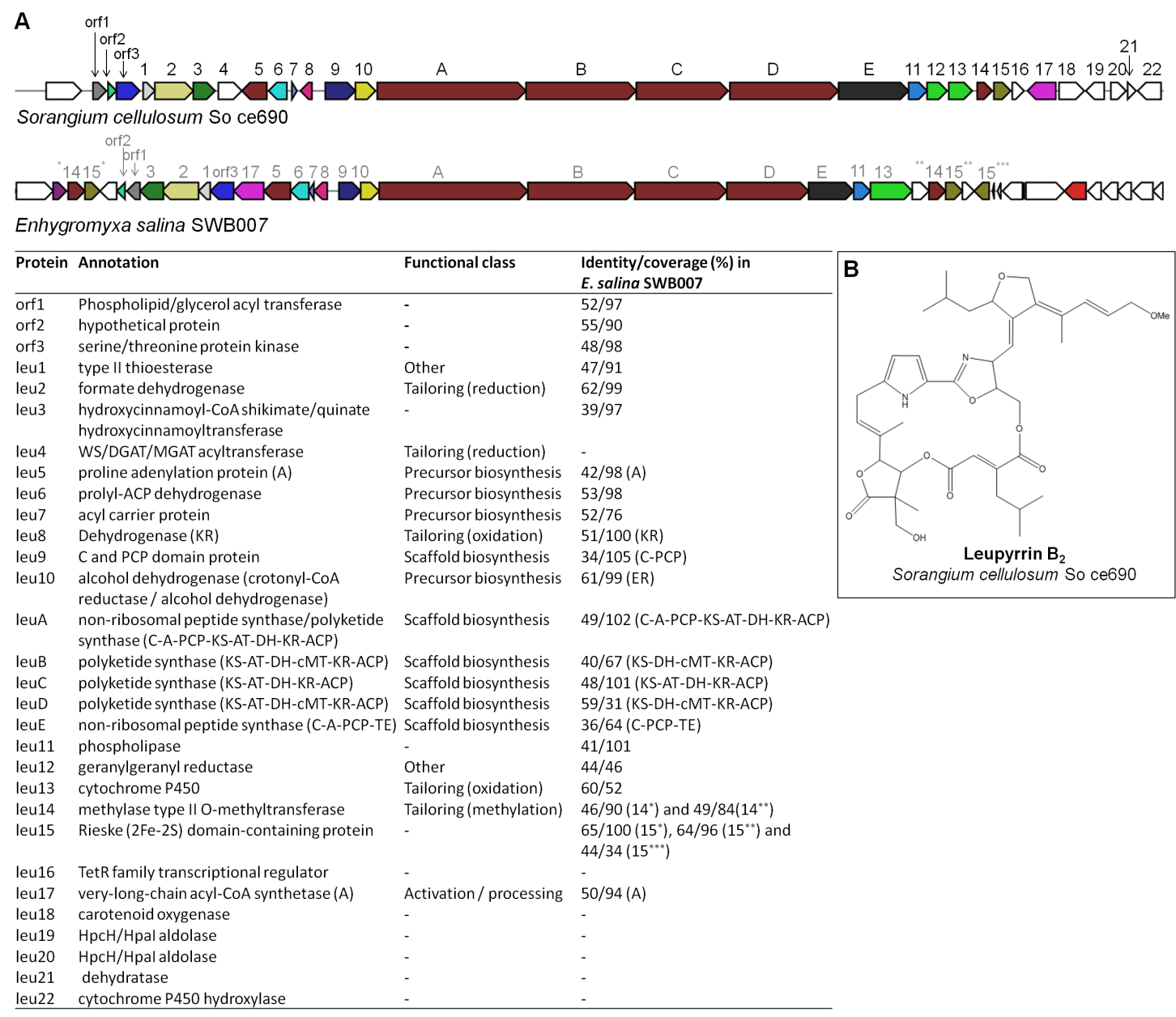


Figure S15. A) Leupyrrin biosynthetic gene cluster in *E. salina* SWB007 and *Sorangium cellulosum.* Proteins encoded in the leupyrrin BGC of the *Sorangium cellulosum* So ce690, their functional class and identity/coverage in *E. salina* SWB007. Same colors/numbers indicate similar annotation. B) Structure of Leupyrrin B_2_ from *Sorangium cellulosum* So ce690.


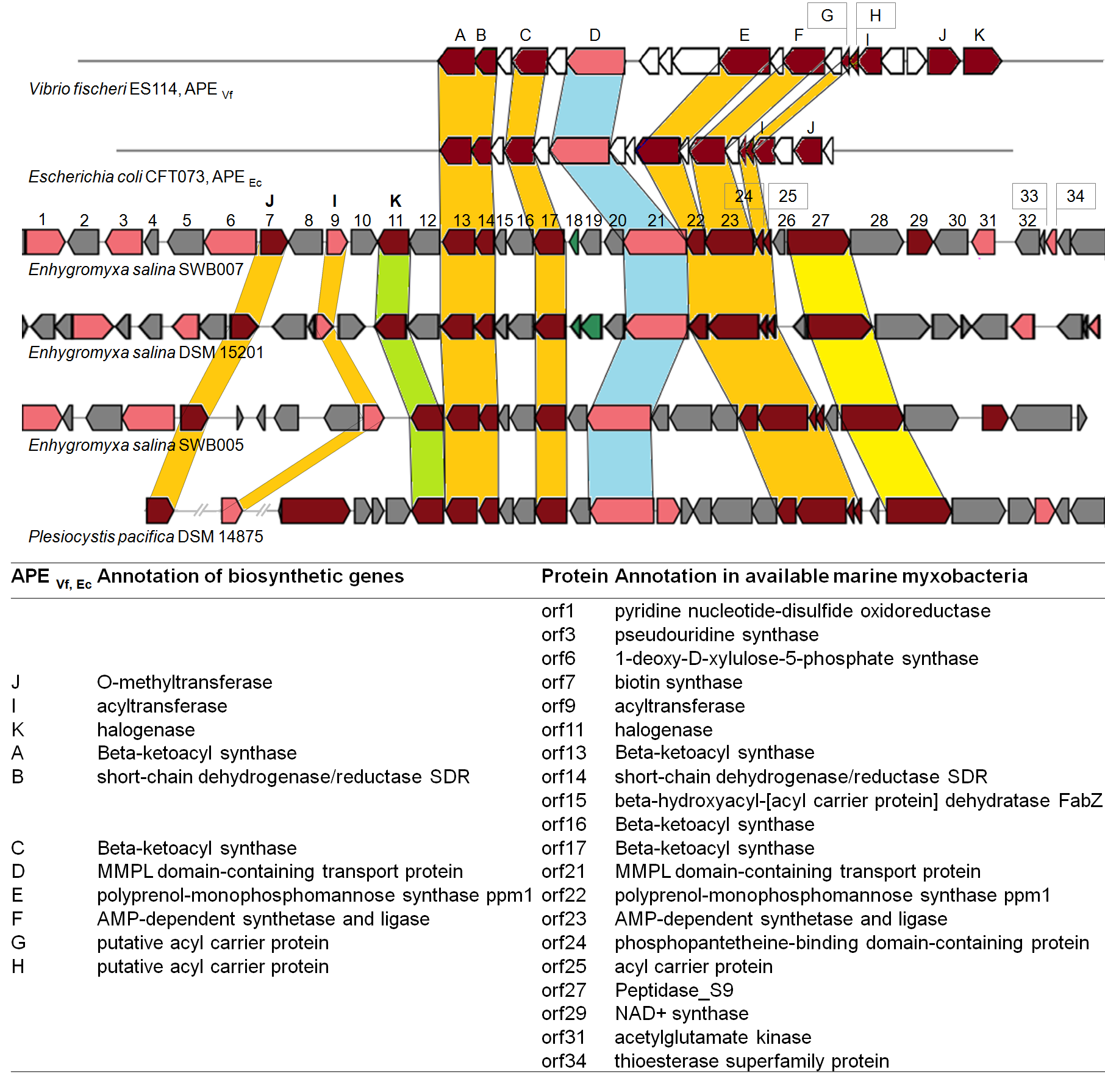


Figure S16. Arylpolyene (APE) BGCs in *E. salina* strains,*P. pacifica* DSM 14875 and the homologous known BGCs of *Vibrio fischeri* ES114 and *Escherichia coli* CFT073. Dark red indicates the biosynthetic genes and pink indicate the transporter genes. Green indicates the regulatory genes. White and grey indicate other genes.


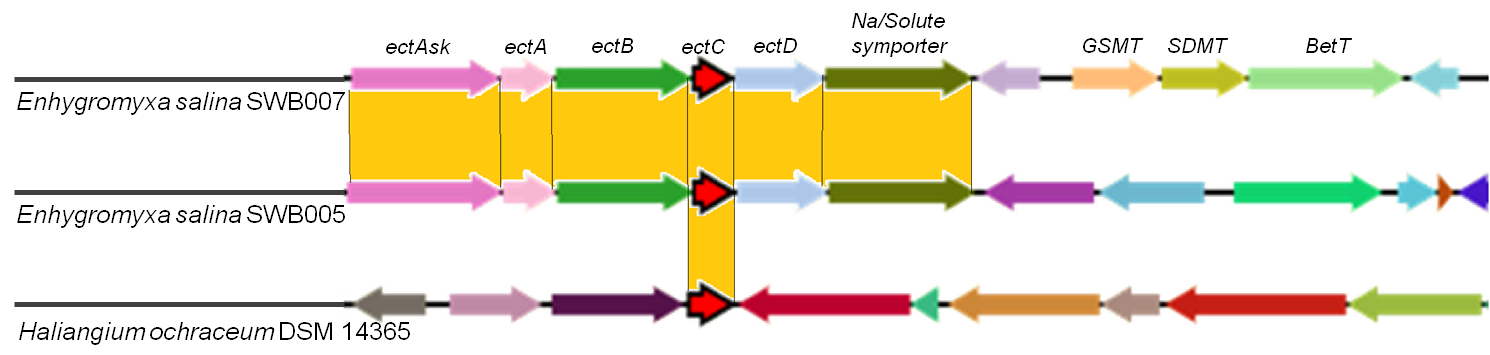


Figure S17. Ectoine/hydroxyectoine BGC in *E. salina* SWB005, *E. salina* SWB007 and *H. ochraceum*. Genes encode for: *ectAsk*: aspartokinase, *ectA*: diaminobutyric acid (DABA) acetyltransferase, *ectB*: DABA aminotransferase, *ectC*: ectoine synthase, *ectD*: ectoine hydroxylase, *Na/solute symporter*, *GSMT*: glycine/sarcosine N-methyltransferase, *SDMT*: sarcosine/dimethylglycine N-methyltransferase, *BetT*: high affinity choline uptake transporter *BetT*. Same colors indicate similar annotation.


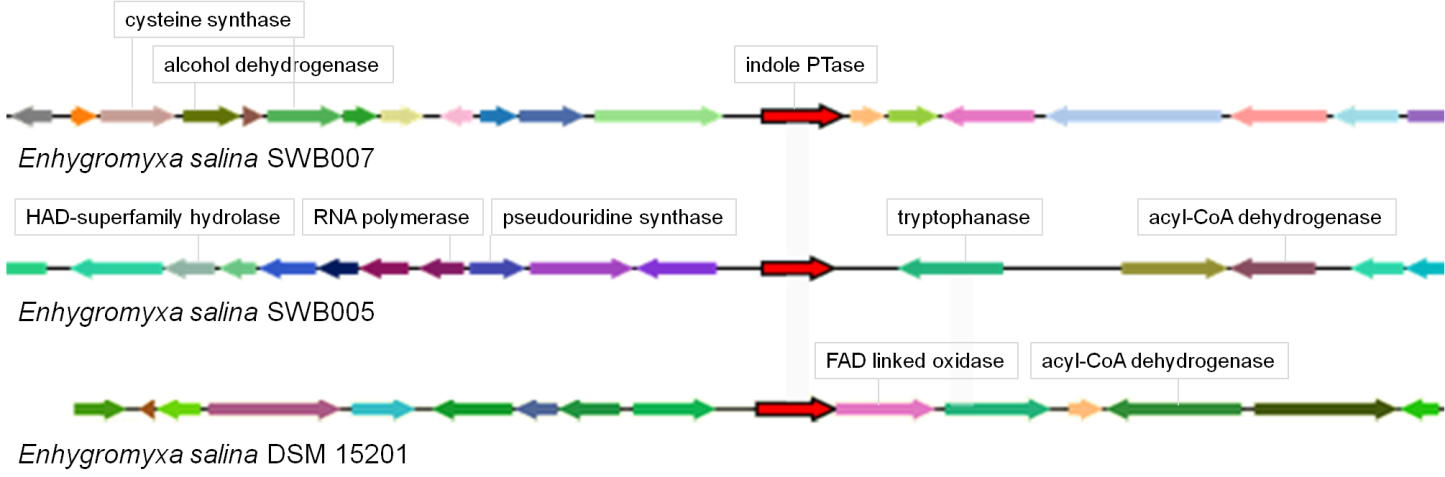


Figure S18. Genomic locus adjacent to the indole prenyltransferase in *E. salina* strains. The same genes have similar color code.


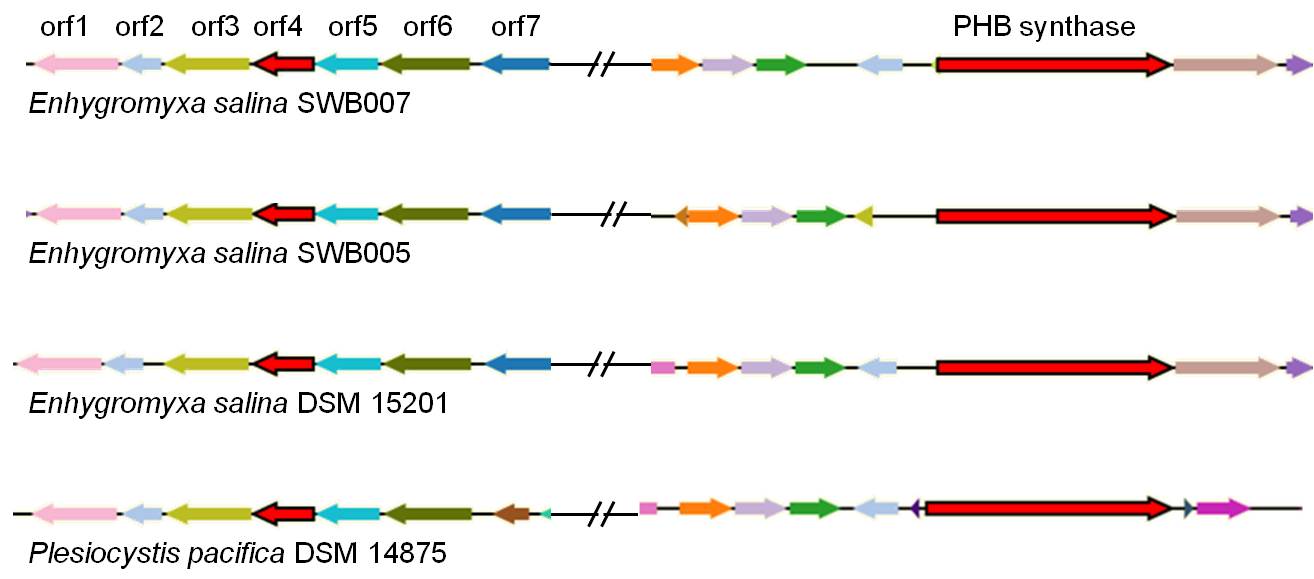


Figure S19. Conserved BGC for polyhydroxybutyrate biosynthesis and production of 3-crotonyl-CoA from 3-hydroxybutyryl-CoA. *orf1*: butyryl-CoA dehydrogenase (EC 1.3.99.2), *orf2*: hypothetical protein, *orf3*: acyl-CoA dehydrogenase, short-chain specific (EC 1.3.99.2), *orf4*: 3-hydroxybutyryl-CoA dehydratase (EC 4.2.1.55), *orf5*: 3-hydroxybutyryl-CoA dehydrogenase (EC 1.1.1.157), *orf6*: acetyl-CoA acetyltransferase (EC 2.3.1.9), *orf7*: alpha/beta hydrolase fold-1 precursor and PHB synthase: polyhydroxybutyrate synthase.


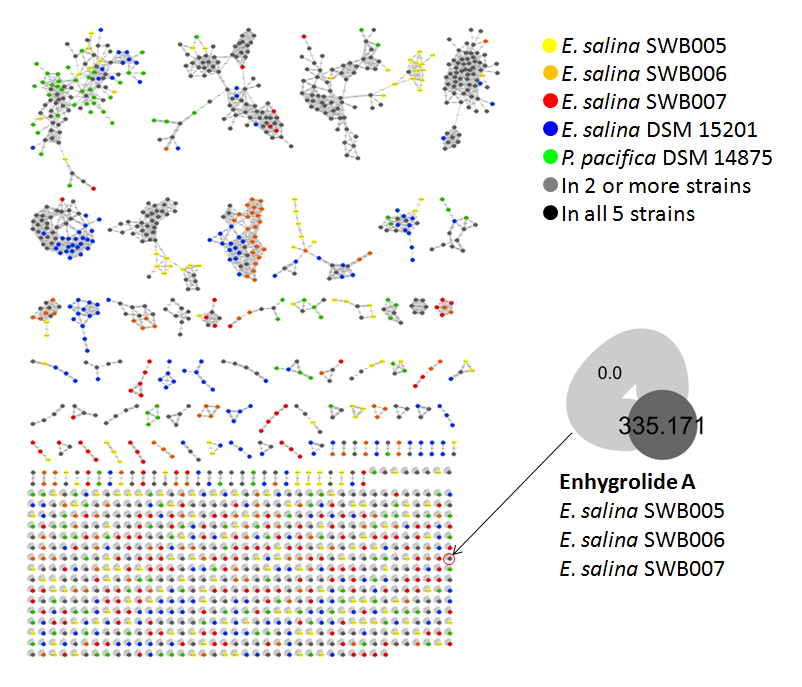


Figure S20. Molecular network of *E.salina* SWB005, SWB006, SWB007, DSM15217 and *P. pacifica* DSM 14875 extracts. Network is color-coded according to detection from single or multiple strains. Enhygrolide A is marked (identified mass was grouped for *E. salina* SWB005, SWB006 and SWB007).

Table S2. ^1^H- and ^13^C-NMR assignments (300 MHz, methanol-d_4_) for enhygrolide A

|  | enhygrolide A |
| --- | --- |

| position | δ_C_ , mult. | δ_H_ (*J* in Hz) |  |
| --- | --- | --- | --- |
| 1 | 170.6, C | - |  |
| 2 | 128.3, C | - |  |
| 3 | 153.3, C | - |  |
| 4 | 149.7, C | - |  |
| 5 | 109.7, CH | 6.38, s |  |
| 6 | 134.7, C | - |  |
| 7 | 131.2, CH | 7,82, d (7.5) |  |
| 8 | 129.6, CH | 7., t (7.5) |  |
| 9 | 129.3, CH | 7.33, t (7.5) |  |
| 10 | 129.6, CH | 7.41, t (7.5) |  |
| 11  12 | 131.2, CH  27.8, CH_2_ | 7.82, d (7.5)  3.67, s |  |
| 13 | 130.4 C | - |  |
| 14  15 | 130.6, CH  116.4, CH | 7.11, d (8.2)  7.82, d (7.5) |  |
| 16 | 156.9, C | - |  |
| 17 | 116.2, CH | 6.75, d (8.2) |  |
| 18 | 130.4, CH | 7.11, d (8.2) |  |
| 19 | 34.2, CH_2_ | 2.58, d (7.5) |  |
| 20 | 30.7, CH | 1.97, m |  |
| 21  22 | 22.9, CH_3_  22.9, CH_3_ | 0.97, d (6.6)  0.97, d (6.6) |  |

Figure S21. ^1^H NMR spectrum of enhygrolide A in Acetone-*d_6_* with traces of enhygrolide B with which enhygrolide A is in a dynamic equilibrium.

Figure S22. ^13^C NMR spectrum of enhygrolide A in Acetone-*d_6_* with traces of the stereoisomer enhygrolide B with which enhygrolide A is in a dynamic equilibrium.

**
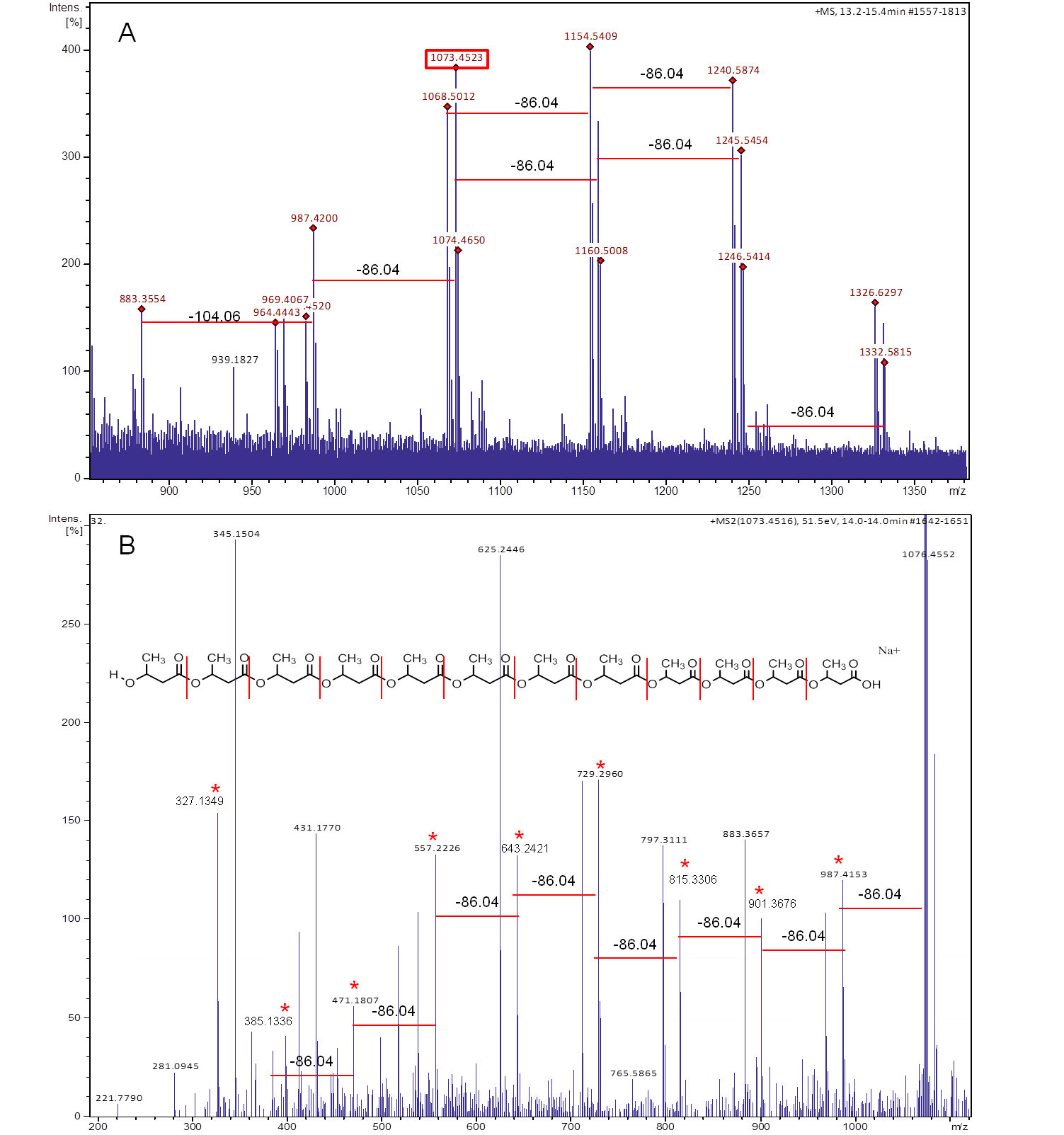
**

Figure S23. A: Mass spectra of different length of polyhydroxybutyric acid (PHB) found in *E. salina* SWB007 with characteristic mass shifts of 86.04 Da. B: MS^2^ spectra of PHB (N=12) with *m/z* = 1073.4516 and characteristic mass shifts of 86.04 Da.


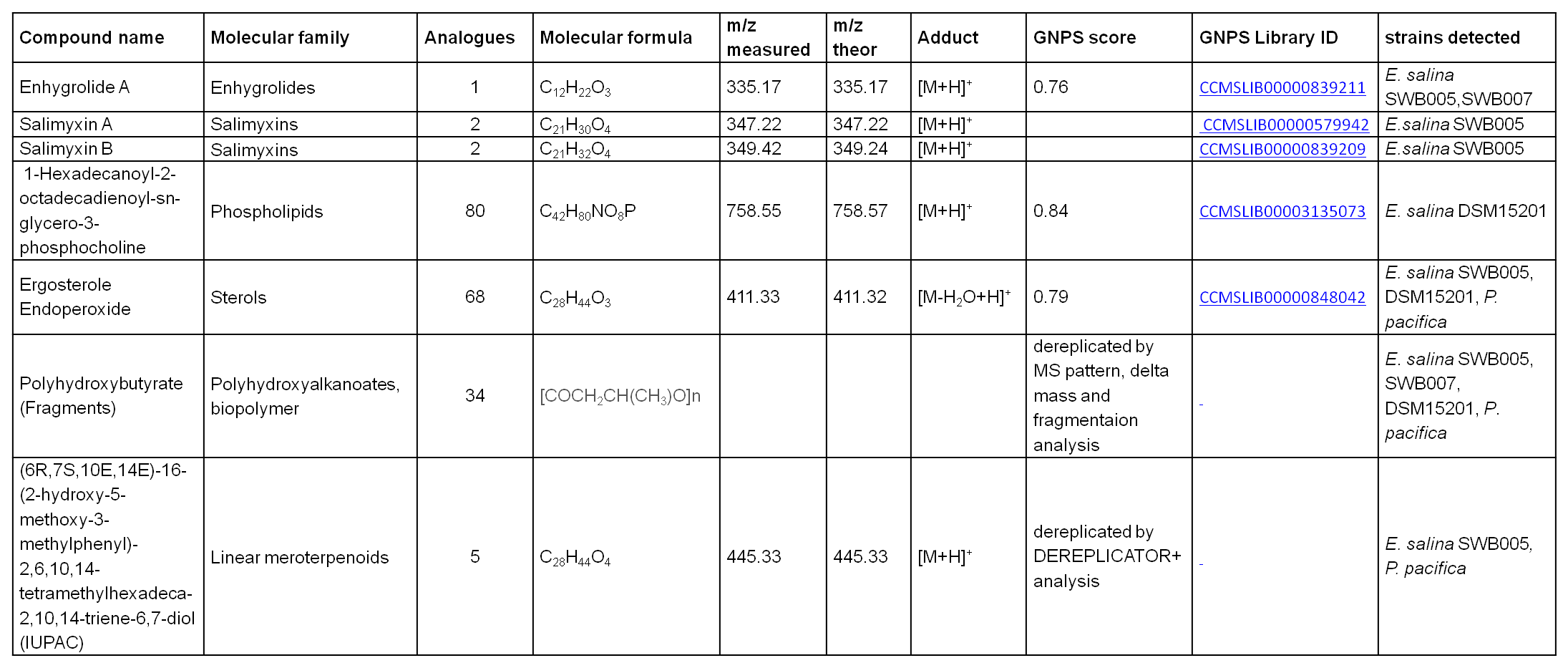


Table 3. Overview of the dereplicated compounds
